# Salt‐inducible kinase 2 confers radioresistance in colorectal cancer by facilitating homologous recombination repair

**DOI:** 10.1002/mco2.70083

**Published:** 2025-01-28

**Authors:** Yuan Meng, Shuo Li, Da‐Shan Lu, Xue Chen, Lu Li, You‐fa Duan, Gao‐yuan Wang, Wenlin Huang, Ran‐yi Liu

**Affiliations:** ^1^ State Key Laboratory of Oncology in South China Guangdong Provincial Clinical Research Center for Cancer Sun Yat‐sen University Cancer Center Guangzhou China; ^2^ Department of Pathology Sun Yat‐sen University Cancer Center Guangzhou China; ^3^ Guangdong Provincial Key Laboratory of Tumor Targeted Drugs & Guangzhou Enterprise Key Laboratory of Gene Medicine Guangzhou DoublleBioproduct Co., Ltd. Guangzhou China

**Keywords:** colorectal cancer, CRISPR‐Cas9 screen, homologous recombination, SIK2

## Abstract

Resistance to radiotherapy remains a critical barrier in treating colorectal cancer (CRC), particularly in cases of locally advanced rectal cancer (LARC). To identify key kinases involved in CRC radioresistance, we employed a kinase‐targeted CRISPR‐Cas9 library screen. This approach aimed to identify potential kinase inhibitors as radiosensitizers. Our screening identified salt‐inducible kinase 2 (SIK2) as a critical factor in CRC radioresistance. Increased SIK2 expression correlated with reduced tumor regression and poorer outcomes in LARC patients undergoing neoadjuvant chemoradiotherapy. The depletion of SIK2 significantly enhanced radiation‐induced apoptosis and tumor regression. Mechanistically, SIK2 interacts with valosin‐containing protein (VCP), promoting its hyperphosphorylation. This modification improves VCP's capacity to extract K48‐linked ubiquitin‐conjugated proteins from chromatin, thus aiding the recruitment of RPA and RAD51 to DNA damage sites. This mechanism strengthens homologous recombination–mediated DNA repair, which contributes to radioresistance. Importantly, ARN‐3236, a SIK2 inhibitor, markedly sensitized CRC cells to radiation both in vivo and in vitro, providing a potential strategy to overcome radioresistance. In summary, our findings reveal a novel mechanism by which SIK2 contributes to the radioresistance of CRC, proposing SIK2 as a potential therapeutic target with its inhibitor significantly enhancing CRC radiotherapy efficacy.

## Graphical abstract

SIK2 promotes hyperphosphorylation of VCP and subsequent extraction of K48–Ub conjugates from DNA damage sites, thereby facilitating homologous recombination and radioresistance. Inhibition of SIK2 with the small molecule ARN‐3236, significantly enhances CRC radiosensitivity by arresting HR‐mediated DNA repair, suggesting a potential therapeutic strategy for overcoming radioresistance in CRC.

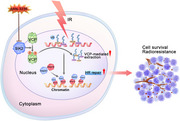

## 1 INTRODUCTION

Colorectal cancer (CRC) is one of the top three cancers globally, with rectal cancer accounting for approximately 30% of these cases.^1^ Locally advanced rectal cancer (LARC), classified as stage II or stage III, is characterized by transmural invasion (T3 or T4) or lymph node metastasis without distant metastasis.^2^ Radiotherapy, a part of neoadjuvant chemoradiotherapy (nCRT), is widely employed for managing advanced CRC, especially LARC. Significant tumor regression following nCRT was associated with improved long‐term outcomes, independent of clinicopathologic parameters.^3^ However, an estimated 15% of patients show no response to nCRT, some even experiencing disease progression.^4^ Patients bearing radioresistant tumors do not gain from nCRT and face its adverse effects, alongside a heightened risk of tumor progression and poor prognosis. To address the challenge of radiotherapy tolerance and enhance treatment outcomes for patients with poor responses to nCRT, it is crucial to uncover the underlying mechanisms of radiotherapy resistance and identify effective therapeutic targets to overcome radioresistance.

As we know, double‐strand breaks (DSBs) are key contributors to ionizing radiation (IR)‐induced cell death. The effectiveness of radiotherapy largely relies on IR‐induced DSBs.^5^, ^6^ Following IR exposure, cells engage two main DSB repair pathways: homologous recombination (HR) and non‐homologous end joining (NHEJ).^7^ Elevated activity in these DSB repair pathways may confer tumors resistant to radiotherapy, as these intrinsic mechanisms are vital for cellular survival after IR.^5^ Consequently, targeting DNA damage repair pathways to confer cancer sensitivity to IR has become a key focus in radiotherapy research.

Kinases, integral to signaling transduction, also play a pivotal role in regulating DSB repair pathways.^8^ Many kinases affect radiosensitivity both directly, by participating in DSB repair, and indirectly, by phosphorylating damage repair‐involved proteins.^8^, ^9^ In addition, as important targets for tumor therapy, many kinases already have corresponding small molecule inhibitors developed.^10^ Therefore, the discovery of new kinases associated with radioresistance in CRC is of great clinical significance, as these kinases could serve as biomarkers for predicting radiotherapy responses and their inhibitors might act as potential radiosensitizers.

In this study, we identified salt‐inducible kinase 2 (SIK2), a serine/threonine kinase of the AMPK subfamily,^11^ as a novel kinase associated with radioresistance in CRC through a kinase‐targeted CRISPR‐Cas9 screen. Elevated SIK2 levels were found to be correlated with reduced tumor regression and poorer prognosis in LARC patients undergoing nCRT. Mechanistically, SIK2 promotes hyperphosphorylation of valosin‐containing protein (VCP) and subsequent extraction of proteins modified with K48‐linked ubiquitin chains (K48–Ub conjugates) from DNA damage sites, thereby facilitating homologous recombination and radioresistance. Furthermore, inhibition of SIK2 with the small molecule ARN‐3236^12^, ^13^, ^14^ significantly enhances CRC radiosensitivity by arresting HR‐mediated DNA repair, suggesting a potential therapeutic strategy for overcoming radioresistance in CRC.

## 2 RESULTS

### 2.1 SIK2 is identified as a radioresistance factor and a prognostic indicator of CRC

**FIGURE 1:**
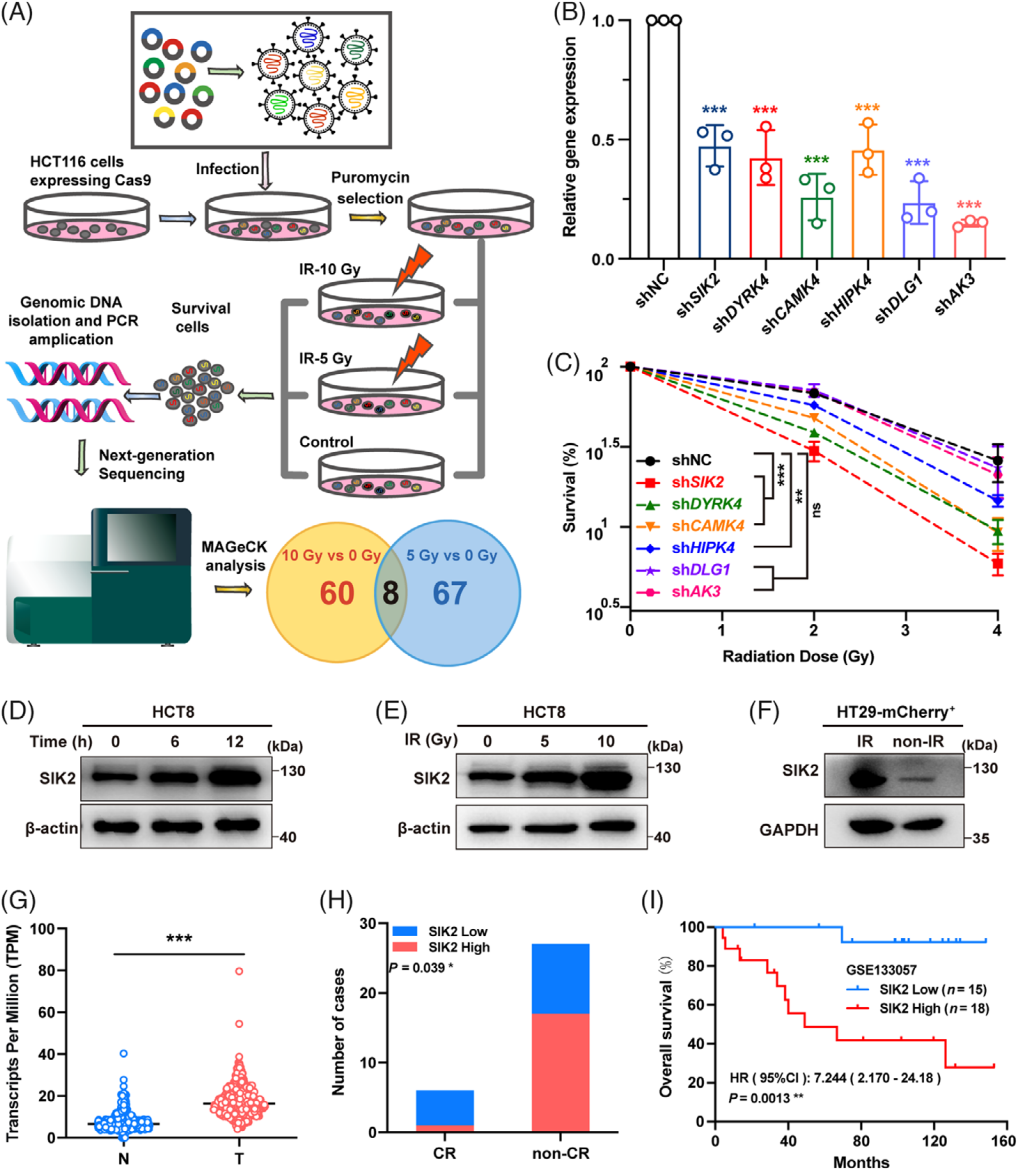
SIK2 is identified as a radioresistance factor and a prognostic indicator of colorectal cancer (CRC). (**A)** The schematic procedure of kinase‐targeted CRISPR‐Cas9 screen that identified eight candidate radioresistance genes. **(B)** Real‐time PCR assay to verify the knockdown efficiency of each candidate gene in HCT116 cells. (**C)** Colony formation assay was used to assess survival after ionizing radiation (IR) in HCT116 cells with stable knockdown of indicated genes. shNC, negative control shRNA, was used as control. Data are represented as mean ± SD of three biological replicates; statistical significance symbols in different colors, compared to the control. (**D** and **E)** Western blot (WB) assays for SIK2 expression at different time points after 10 Gy IR (**D**) or 12 h after different doses of IR (**E**). **F,** WB assay of SIK2 protein in HT29‐mCherry^+^ cells derived from xenografts with IR and without ionizing radiation (non‐IR). (**G)** SIK2 expression in colorectal tumor (T) and normal tissues (N). SIK2 expression was analyzed using RNAseq data from The Cancer Genome Atlas (TCGA) and the Genotype‐Tissue Expression (GTEx) database. **(H** and **I)** Analysis of a published CRC cohort (GSE133057). The cut‐off value for SIK2 level was deduced according to overall survival (OS) using a receiver operating characteristic (ROC) curve, and patients were categorized into two groups based on SIK2 level (high or low). (**H)** Distribution of SIK2 expression levels in patients with complete (CR) or non‐complete response (non‐CR) to nCRT. Tumor response to nCRT was evaluated by the tumor regression grade (TRG), which was proposed in the 8th Edition of the American Joint Committee on Cancer (AJCC) Staging Manual System. TRG 0 was considered as CR, TRG 1–3 was considered as non‐CR. (**I)** OS curves according to high or low SIK2 expression levels. ns, not significant; *p < 0.05; **p < 0.01; ***p < 0.001.

To identify radioresistance factors in CRC, we performed a kinase‐targeted CRISPR‐Cas9 screen using the Dharmacon Edit‐R Human Lentiviral Protein Kinases sgRNA Arrayed Library containing 2613 sgRNAs targeting 697 kinase genes. HCT116 cells stably expressing Cas9 were infected with lentiviral sgRNAs, selected with puromycin, and subsequently exposed to various X‐ray doses. Surviving cells were sorted, and sgRNA copy numbers were analyzed through next‐generation sequencing and MAGeCK‐negative selection (Figure 1A). As a result, 68 and 75 candidates with depleted sgRNA copy numbers were selected from the surviving cells after 10 or 5 Gy X‐ray treatment (Tables S1 and S2), and eight genes, *HIPK4, CLK2, SIK2, CAMK4, AK3, DLG1*, *DYRK4*, and *CALM2*, were thus identified by intersecting these two subsets as candidate radioresistance factors (Figure 1A). Since *CLK2* and *CALM2* have been reported to be associated with radioresistance by regulating DNA damage repair,^15^, ^16^ subsequent studies have focused on the remaining six candidate genes. The fold change of sgRNA copy numbers targeting these six candidate radioresistant genes is shown in Figure S1A. To confirm the result of this screen, we first constructed HCT116 cells with stable knockdown of each candidate gene and confirmed the knockdown efficiency (Figure 1B). Subsequently, colony formation assays after X‐ray exposure were performed in these cells with stable knockdown. The results showed that knockdown of *SIK2*, *DYRK4*, *CAMK4*, and *HIPK4* significantly reduced the radioresistance in HCT116 cells, and the cells with *SIK2* knockdown were the most sensitive to X‐rays (Figure 1C, Figure S1B). In addition, IR led to a significant increase in SIK2 protein levels in CRC cells, which was time‐ and dose‐dependent (Figure 1D,E). Similarly, SIK2 protein levels were remarkably higher in HT29‐mCherry xenografts exposed to multiple low doses of IR than in xenografts without IR (Figure S1C, Figure 1F), underscoring SIK2's critical role in CRC radioresistance.

We also assessed the expression of SIK2 and clinical relevance in CRC. SIK2 expression was significantly higher in tumor cells and tissues than in normal counterparts (Figure S1D,E, Figure 1G). Analysis of CRC patient data (GSE17536) showed that elevated SIK2 expression correlates with reduced overall survival (OS), with a hazard ratio (HR) of 2.497 (*p* = 0.005; Figure S1F). Critically, among LARC patients who received nCRT, the proportion of patients with high SIK2 expression in pre‐treatment biopsies in the incomplete tumor regression group was significantly higher than that in the complete tumor regression group (GSE133057; Figure 1H). Survival analysis further revealed that high SIK2 expression was associated with decreased OS post‐nCRT (HR = 7.244, *p* = 0.0013; Figure 1I). Thus, these findings indicate that SIK2 confers radioresistance in CRC and significantly correlates with the therapeutic outcomes of nCRT.

### 2.2 SIK2 enhances the radioresistance of CRC both in vitro and in vivo

**FIGURE 2:**
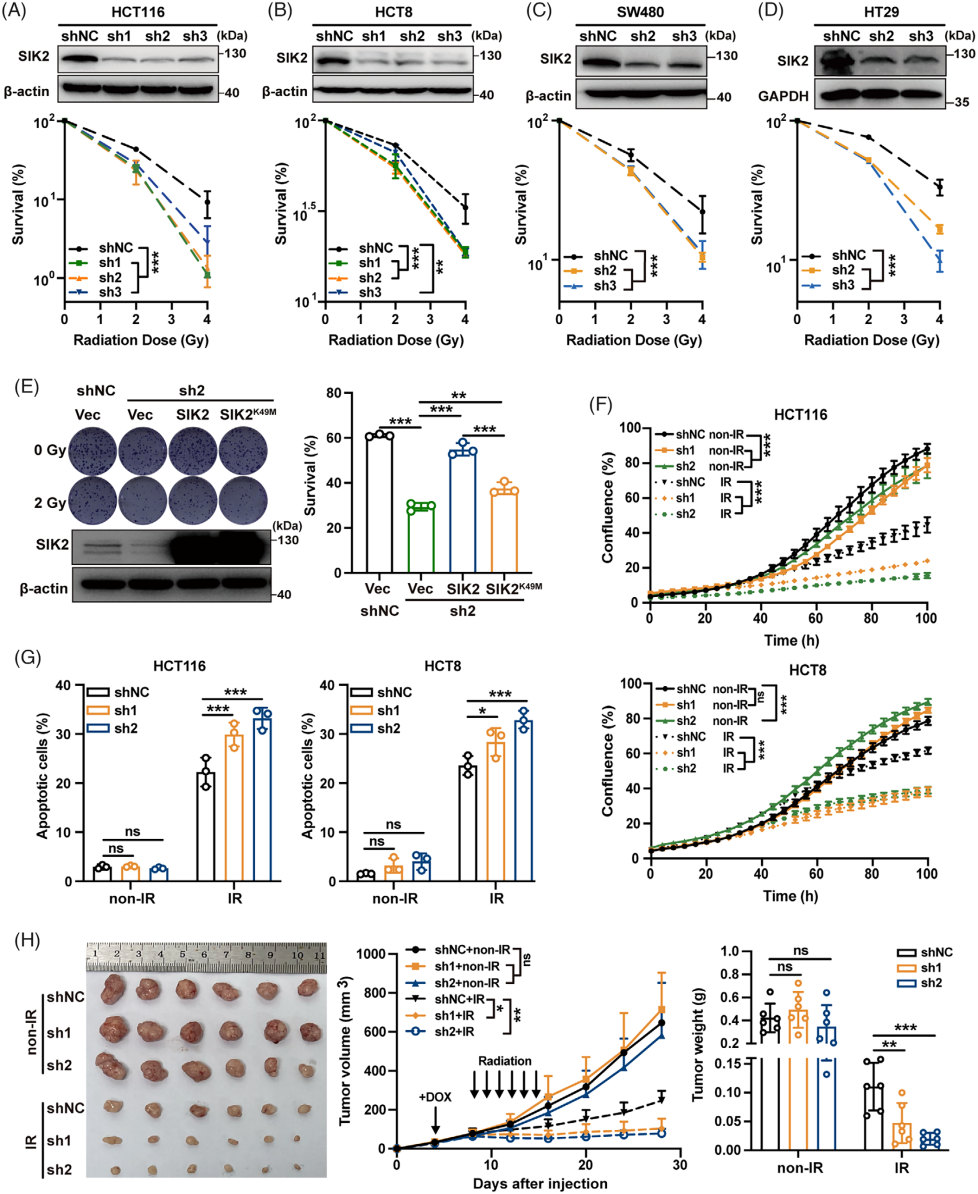
SIK2 promotes the radioresistance of colorectal cancer (CRC). (**A–D)** Colony formation assays were used to assess survival after ionizing radiation (IR) for HCT116 (**A**), HCT8 (**B**), SW480 (**C**), and HT29 (**D**) cells with SIK2 knockdown. Upper, western blot (WB) pictures; lower, survival rate after exposure to different doses of X‐ray. (**E)** Reintroducing SIK2 or a kinase‐dead mutant SIK2 (SIK2^K49M^) in HCT116 cells that were knockdown for endogenous SIK2 with sh2, a shRNA targeting the SIK2 3′‐UTR, and the survival of these cells after IR was examined by colony formation assays. Left: representative colony formation pictures and WB pictures, right: the survival rates after IR are represented as mean ± SD of three biological replicates. Vec, lentiviral vector; SIK2, lentiviral vector expressing SIK2; SIK2^K49M^, lentiviral vector expressing a kinase‐dead mutant of SIK2. (**F)** Effects of SIK2 knockdown on the cell proliferation of HCT116 and HCT8 cells after IR analyzed by the IncuCyte S3 Live‐Cell Analysis System. Data are represented as mean ± SEM (*n* = 8). (**G)** Assessment of SIK2 knockdown on apoptosis in HCT116 and HCT8 cells 48 h after IR by flow cytometry‐based annexin V/PI double‐staining assays. The percentages of apoptotic cells are represented as mean ± SD of three biological replicates. (**H)** Effects of SIK2 knockdown (dox‐inducible) on radiotherapy efficacy in the subcutaneous xenograft model. Left, removed xenografts; middle, tumor volume; right, tumor weight; data are represented as mean ± SD (*n* = 6). non‐IR, without ionizing radiation. shNC, negative control; sh1‐3, *SIK2* shRNAs. ns, not significant; *p < 0.05; **p < 0.01; ***p < 0.001.

To validate SIK2's role in radioresistance in CRC, we performed colony formation assays on CRC cells with either stable SIK2 knockdown or overexpression, following X‐ray exposure. These assays revealed that SIK2 knockdown significantly impaired colony formation in irradiated CRC cells (Figure 2A–D, Figure S2A), with sensitivity enhancement ratios greater than 1 in all knockdown groups (Table S3). Conversely, SIK2 overexpression notably enhanced colony formation (Figure S2B). Importantly, reintroducing SIK2 expression in SIK2‐knockdown HCT116 cells substantially restored the impaired colony formation after X‐ray exposure, whereas a kinase‐dead mutant^17^ of SIK2 (SIK2^K49M^) only marginally restored this function (Figure 2E), suggesting that the kinase activity of SIK2 is crucial for its function in promoting radioresistance. Real‐time monitoring with the IncuCyte system showed that SIK2 knockdown led to a significant decrease in cell proliferation after X‐ray exposure (Figure 2F). Annexin V/PI double‐staining and western blot analyses further demonstrated that SIK2 knockdown enhanced IR‐induced apoptosis in HCT116 and HCT8 cells (Figure 2G, Figure S2C,D). For further investigation, we used HCT116 cells with a Tet‐On inducible SIK2 knockdown system to establish subcutaneous CRC xenografts followed by doxycycline (DOX) induction and radiotherapy (X‐rays 2 Gy/day for 7 days). Xenografts with SIK2 knockdown (HCT116‐sh1/sh2) exhibited a significant reduction in size and weight compared to control xenografts (HCT116‐shNC) after radiotherapy (Figure 2H). Immunohistochemistry (IHC) analysis showed decreased KI67 staining, a cell proliferation marker, in CRC xenografts with SIK2 knockdown following radiotherapy (Figure S2E). Additionally, the radiotherapy effect, represented as tumor inhibition rate, was markedly enhanced in the HCT116‐sh1/sh2 groups compared to in the HCT116‐shNC group (90.38% and 94.25% vs. 73.85%). These results collectively confirm that SIK2 enhances CRC cell survival from IR by promoting cell proliferation and inhibiting IR‐induced apoptosis, thereby contributing to radioresistance in both in vitro and in vivo models.

### 2.3 SIK2 mediates radioresistance by facilitating HR‐mediated DNA repair in CRC cells

**FIGURE 3:**
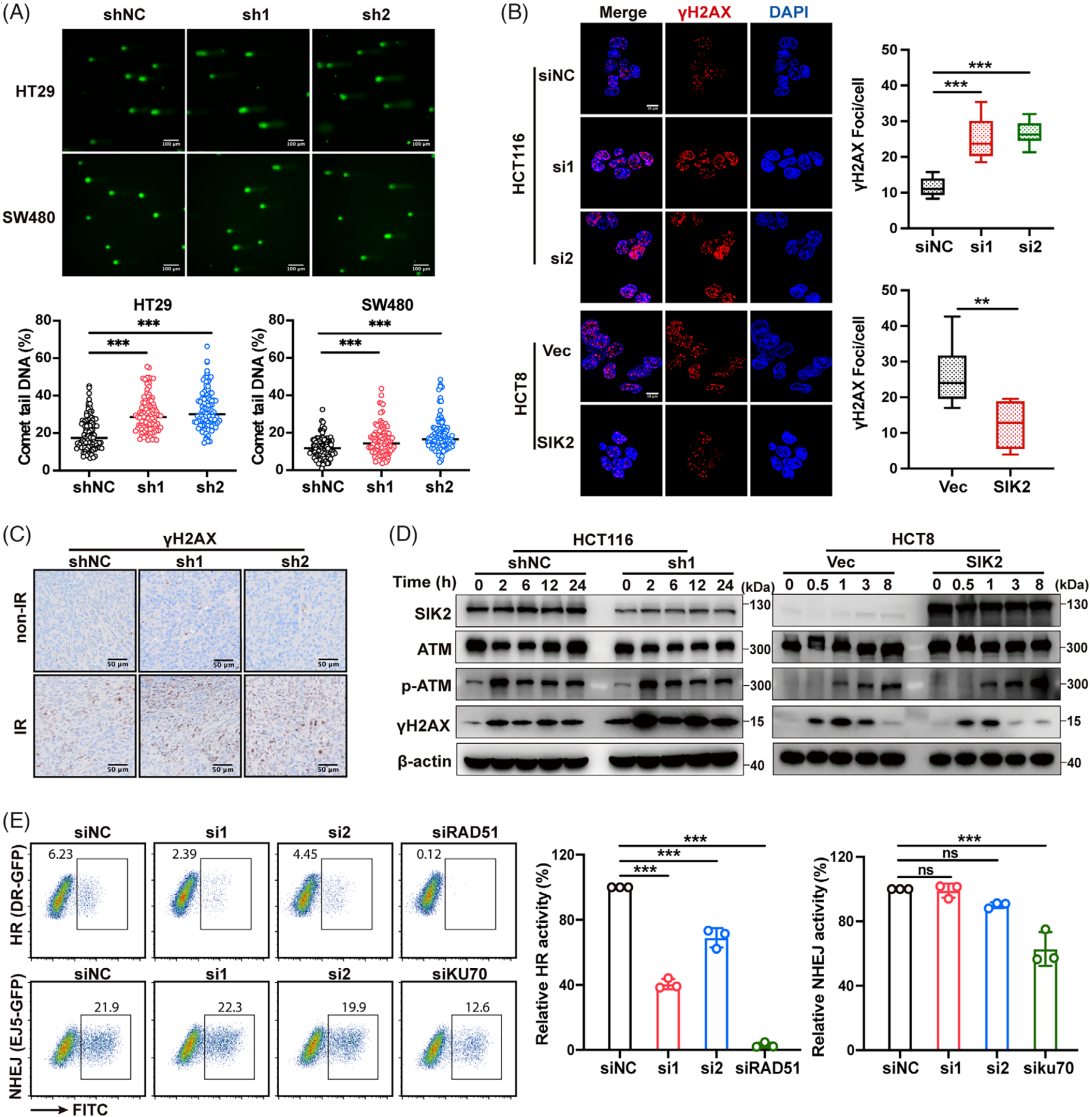
SIK2 promotes radioresistance by facilitating HR. (**A)** Comet assay was used to assess DNA damage in colorectal cancer (CRC) cells 2 h after treatment with ionizing radiation (IR). Upper, representative pictures, scale bar, 100 µm; lower, spot charts indicating the percent of comet tail DNA, at least 100 cells were counted in each condition. (**B)** Immunofluorescence was used to assess the effect of SIK2 expression on γH2AX foci at 2 h after treatment with X‐rays (HCT116 cells were exposed to 6 Gy X‐ray, HCT8 cells were exposed to 12 Gy X‐ray). Left, representative immunofluorescence images, scale bar, 10 µm; right, boxplots indicating the γH2AX foci numbers per cell, at least 100 cells were counted in each condition. **(C)** Representative images of IHC staining for γH2AX in the harvested xenografts. Magnification, × 40. Scale bar, 50 µm. non‐IR, without ionizing radiation. (**D)** Western blot (WB) assays of indicated proteins in CRC cells. Proteins were harvested at indicated time points post‐IR from CRC cells with SIK2 knockdown (HCT116) and overexpression (HCT8). (**E)** Direct‐repeat‐green fluorescent protein (DR‐GFP) (HR) and EJ5‐GFP (NHEJ) reporter assays were conducted to assess the effects of SIK2 knockdown on DSB repair (left, representative pictures of flow cytometry; right, the relative HR or NHEJ activity); data are represented as mean ± SD of three biological replicates. shNC, negative control; sh1‐2, *SIK2* shRNAs; siNC, negative control; si1‐2, *SIK2* siRNAs; siRAD51 and siKU70, siRNAs targeting *RAD51* and *KU70* were used as positive controls. Vec, lentiviral vector; SIK2, lentiviral vector expressing SIK2. ns, not significant; **p < 0.01; ***p < 0.001.

IR inflicts severe DNA damage, notably DSBs, which can lead to cell death or mutations if not properly repaired.^6^ To assess the influence of SIK2 on DSB levels, we utilized the comet assay. In the absence of IR, SIK2 knockdown cells showed a slight increase in tail DNA compared to the control group (Figure S3A). While following IR, this increase became more pronounced in the SIK2 knockdown cells (Figure 3A). The accumulation of phospho‐H2AX (γH2AX), a hallmark of DNA damage, was assessed via immunofluorescence (IF) and IHC assays. The results indicated that SIK2 knockdown alone led to a mild increase in γH2AX foci in unirradiated cells (Figure S3B). After radiation, SIK2‐silenced HCT116 cells exhibited a substantial increase in γH2AX foci compared to control cells, whereas SIK2‐overexpressing HCT8 cells had significantly fewer foci than parental cells post‐IR (Figure 3B). Similarly, SIK2 silencing resulted in increased staining of γH2AX in CRC xenografts assessed by IHC (Figure 3C). We also examined γH2AX kinetics after irradiation. γH2AX levels remained elevated in SIK2‐knockdown HCT116 cells across all time points (Figure 3D), indicating persistent DNA damage signaling. In contrast, γH2AX levels declined more rapidly in SIK2‐overexpressing HCT8 cells (Figure 3D), indicating a potential role for SIK2 in DSB repair facilitation.

Ataxia‐telangiectasia mutated (ATM) kinase, an early responder to DNA damage, was phosphorylated (p‐ATM) following X‐ray exposure.^8^ However, changes in SIK2 expression did not affect p‐ATM levels, indicating that SIK2 may not influence the initial DNA damage response but rather modulates subsequent repair processes (Figure 3D). DSBs trigger a checkpoint system that leads to G2/M arrest, providing time for DNA repair.^6^ Cell cycle analysis revealed that SIK2 knockdown prolonged G2/M arrest after radiation (Figure S3C), suggesting compromised DNA repair and sustained cell cycle arrest.

To further explore SIK2's involvement in DSB repair pathways, we utilized the direct‐repeat‐green fluorescent protein (DR‐GFP) and end‐joining 5‐GFP (EJ5‐GFP) reporter assays^18^ to assess HR and NHEJ activities. The results showed that SIK2 knockdown significantly reduced HR activity without affecting NHEJ activity (Figure 3E, Figure S3D,E). Collectively, these results indicate that SIK2 promotes HR‐mediated DSB repair, thereby contributing to radioresistance in CRC.

### 2.4 SIK2 interacts with VCP, inducing its hyperphosphorylation post‐irradiation and leading to radioresistance in CRC

**FIGURE 4:**
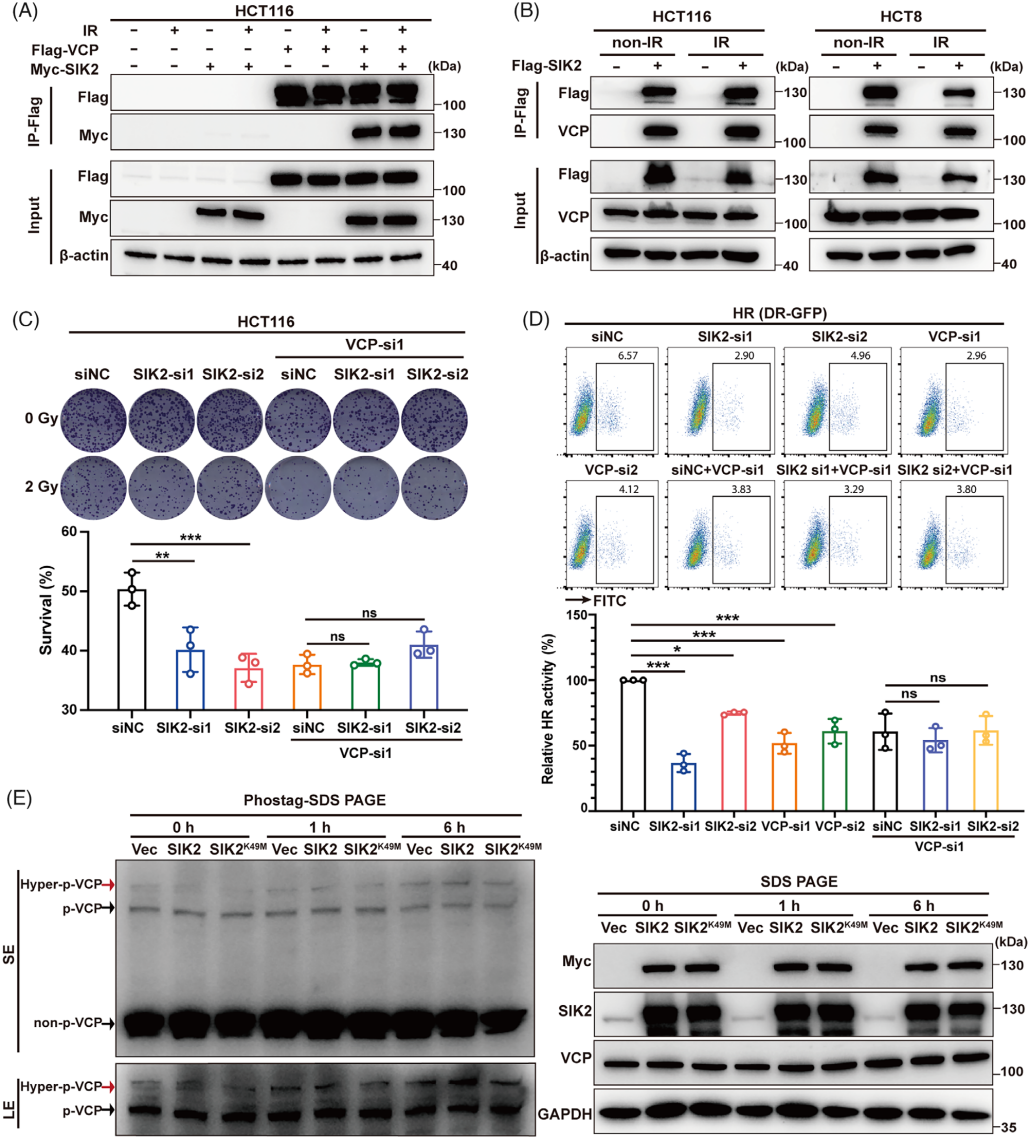
SIK2 facilitates hyperphosphorylation of VCP, thereby promoting radioresistance in colorectal cancer. **A** and **B**, HCT116 cells transiently transfected of indicated expression plasmids (**A**); HCT116 and HCT8 cells stable overexpressing Vec or Flag‐SIK2 (**B**). Cells were treated with ionizing radiation (IR) (0 or 12 Gy) and harvested for Co‐IP and western blot (WB) assays. Flag‐VCP, lentiviral vector expressing valosin‐containing protein (VCP) with Flag tag; Myc‐SIK2, lentiviral vector expressing SIK2 with Myc tag; Flag‐SIK2, lentiviral vector expressing SIK2 with Flag tag. non‐IR, without ionizing radiation. (**C** and **D)** Colony formation assay (upper, representative colony formation pictures; lower, the survival rate post‐IR: **C**) and direct‐repeat‐green fluorescent protein (DR‐GFP) (HR) reporter assay (upper, representative pictures of flow cytometry; lower, the relative HR activity: **D**) were used to assess radiosensitivity and HR activity under SIK2 transient knockdown or VCP transient knockdown or a transient combination knockdown of both. Data are represented as mean ± SD of three biological replicates. (**E)** WBs (left, Phos‐tag‐SDS PAGE; right, SDS PAGE) of the cell lysates at different time points post‐IR (12 Gy) from HCT116 cells expressing Vec (lentiviral vector), SIK2 (lentiviral vector expressing SIK2), and SIK2^K49M^ (lentiviral vector expressing a kinase‐dead mutant of SIK2). LE, long‐term exposure; SE, short‐term exposure; Hyper‐p‐VCP, hyperphosphorylated VCP; p‐VCP, phosphorylated VCP; non‐p‐VCP, non‐phosphorylated VCP. ns, not significant; *p < 0.05; **p < 0.01; ***p < 0.001.

It has been reported that SIK2 inhibition repressed the expression of DNA repair genes, such as FANCD2, EXO1, and XRCC4 in ovarian and triple‐negative breast cancers.^12^ However, our mRNA sequencing data revealed that SIK2 knockdown did not alter the transcription of these genes in HCT116 cells, either before or after radiation exposure (Figure S4). This suggests that SIK2 may enhance HR through alternative mechanisms. Given that previous data suggested SIK2's promotion of radioresistance is predominantly driven by its kinase activity, we conducted mass spectrometry and immunoprecipitation (IP) experiments to identify downstream molecules that interact with SIK2 and may be phosphorylated by SIK2. Among the proteins interacting with SIK2, VCP emerged as a protein of interest (Figure S5A), which has been reported to facilitate DSB repair by removing K48–Ub conjugates from sites of DNA damage.^19^, ^20^ IP‐western blot analyses revealed that SIK2 (Myc‐SIK2 or endogenous SIK2) was pulled down by VCP (FLAG‐VCP or endogenous VCP) in lysates of both radiation‐treated and ‐untreated HCT116 cells (Figure 4A, Figure S5B). VCP was also pulled down by SIK2 (FLAG‐SIK2 or endogenous SIK2) in HCT116 and HCT8 CRC cells (Figure 4B, Figure S5B).

To further elucidate the role of the SIK2‐VCP interaction, we assessed the effects of SIK2 on HR activity and radiosensitivity after silencing VCP. The results showed that VCP knockdown almost completely abolished the SIK2‐induced increase in HR activity and radioresistance in HCT116 cells (Figure 4C,D, Figure S5C,D), suggesting that SIK2's promotion of DSB repair in CRC is dependent on its interaction with VCP.

Due to the lack of specific antibodies for phospho‐VCP, we used SuperSep Phos‐tag polyacrylamide gels^21^ to detect whether SIK2 promotes VCP phosphorylation after they interacted with each other. Our results showed that radiation can induce an increase in phosphorylation of VCP, with a significant rise in hyperphosphorylation (at least two or more sites being phosphorylated). Notably, 6‐h post‐radiation, the hyperphosphorylation of VCP was remarkably increased in HCT116 cells overexpressing SIK2 other than kinase‐dead mutant (SIK2^K49M^) (Figure 4E). In conclusion, our data suggest that SIK2 enhances radioresistance in CRC cells by binding to VCP and promoting the hyperphosphorylation of VCP following X‐ray exposure.

### 2.5 SIK2 promotes the recruitment of RPA and RAD51 to chromatin via K48–Ub conjugates extraction driven by VCP

**FIGURE 5:**
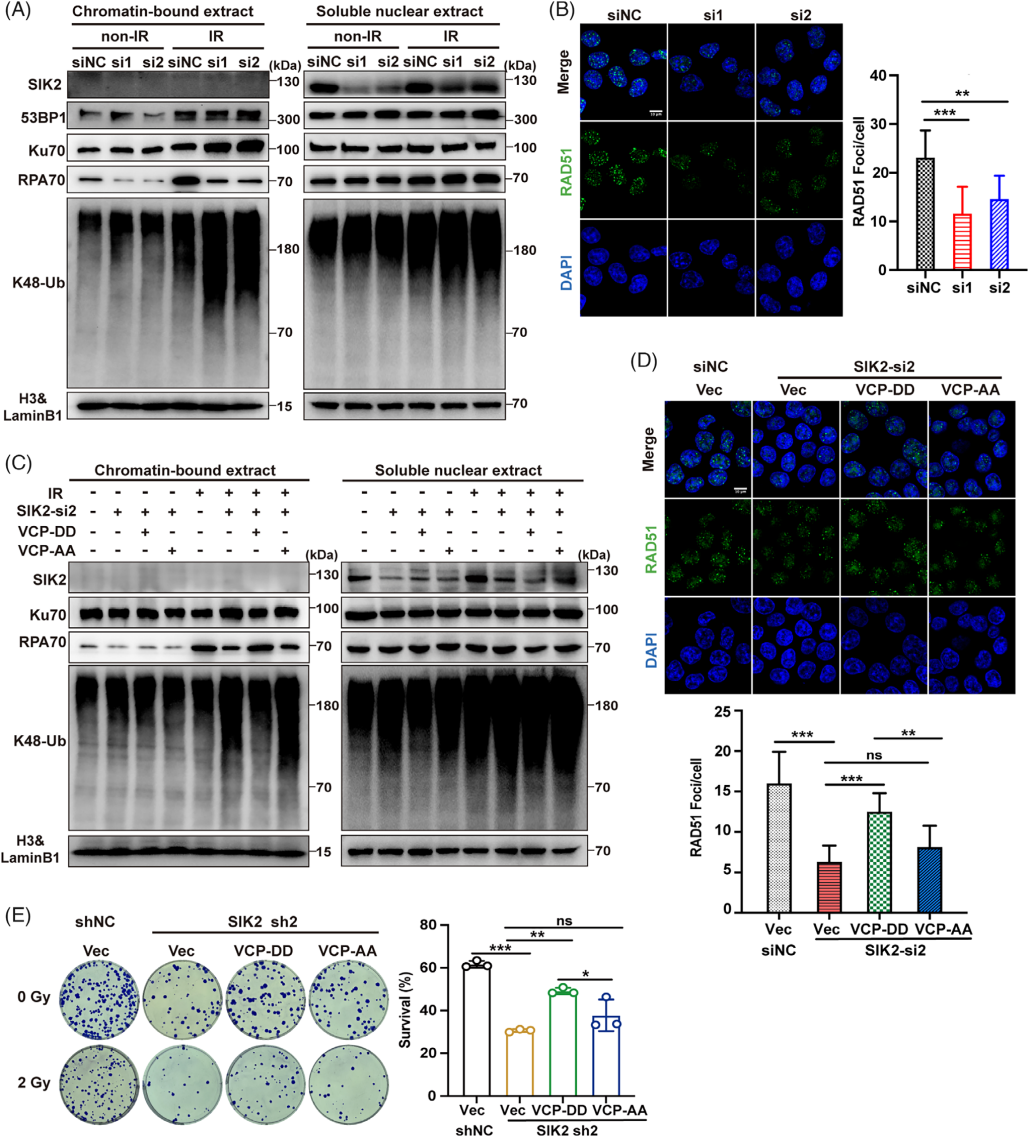
SIK2 promotes homologous recombination repair via K48–Ub conjugates extraction driven by valosin‐containing protein (VCP). (**A)** Western blots (WBs) of the indicated proteins in subcellular fractions of HCT116 cells transiently transfected of siNC and SIK2 siRNAs 6 h after ionizing radiation (IR) (12 Gy); non‐IR, without ionizing radiation. (**B)** Immunofluorescence was used to assess the effect of SIK2 knockdown on RAD51 foci 8 h after IR (12 Gy). Left, representative immunofluorescence images, scale bar, 10 µm; right, foci numbers are presented as the mean ± SD, at least 100 cells were counted in each condition. siNC, negative control; si1‐2, *SIK2* siRNAs. (**C–E)** HCT116 cells with endogenous SIK2 silenced by siRNA or shRNA, followed by restoration of VCP‐DD or VCP‐AA expression. WBs of the indicated proteins in subcellular fractions (**C**). Immunofluorescence was used to assess RAD51 foci 8 h after treatment with 12 Gy X‐rays (**D**). Upper, representative immunofluorescence images, scale bar, 10 µm; lower, foci number is presented as the mean ± SD, at least 100 cells were counted in each condition. Colony formation assays were used to assess survival after exposure to X‐rays (**E**). Left, representative colony formation pictures; right, the survival rate is presented as the mean ± SD of three biological replicates. ns, not significant; *p < 0.05; **p < 0.01; ***p < 0.001.

To further verify whether SIK2 promotes HR repair via K48–Ub conjugates extraction driven by VCP, we performed subcellular fraction analysis to examine the chromatin recruitments of key DSB repair‐related proteins. The results showed that SIK2 depletion led to a modest rise in K48‐Ub conjugates in cytoplasmic and soluble nuclear fractions, yet a marked increase within chromatin‐bound extracts (Figure 5A, Figure S6A), suggesting an impaired capability of VCP to remove these conjugates from chromatin under SIK2‐deficient conditions. Given the role of VCP in extracting the Ku70/80 rings from chromatin and enabling 53BP1 chromatin accumulation,^22^, ^23^ we discovered that SIK2 knockdown resulted in abnormal retention of Ku70 on chromatin, while 53BP1 recruitment remained unaltered (Figure 5A). What is more, research has shown that VCP not only extracts Ku70/80 rings sterically trapped after DNA repair but also removes part of Ku before ligation from unrepaired DNA ends, thereby affecting repair pathway choice to favor HR over NHEJ repair.^22^ Correspondingly, our results showed a significant reduction in chromatin‐bound RPA70, while RPA70 protein levels in other extracts were unaffected (Figure 5A, Figure S6A). Immunofluorescenceassays corroborated these findings, revealing a reduction in RAD51 foci in SIK2‐silenced cells (Figure 5B), indicating an impaired HR process due to ineffective K48‐Ub conjugate extraction, abnormal persisting Ku trapped on chromatin, and subsequent impaired HR repair proteins recruitment.

Preliminary data indicated that post‐IR, SIK2 enhances VCP hyperphosphorylation, with phosphorylation at serine 770 and 784 known to facilitate VCP function significantly.^24^, ^25^ We investigated if SIK2 facilitates the removal of chromatin‐bound K48‐Ub conjugates through VCP hyperphosphorylation. Post‐irradiation nuclear translocation of SIK2 was observed (Figure 5A,C), supporting previous findings. Crucially, a VCP mimicking constitutive phosphorylation (S770D S784D; VCP‐DD) mutant but not the non‐phosphorylatable mutant (S770A S784A; VCP‐AA) decreased the chromatin accumulation of K48‐Ub conjugates and largely reversed the decrease in chromatin‐bound RPA70 and RAD51 foci induced by SIK2 knockdown (Figure 5C,D, Figure S6B). Colony formation assays further demonstrated that the VCP‐DD mutant, unlike the non‐phosphorylatable VCP‐AA mutant, significantly restored radioresistance in SIK2‐knockdown cells (Figure 5E). These results collectively suggest that SIK2 may promote VCP hyperphosphorylation and facilitate VCP to extract chromatin K48‐Ub conjugates such as K48‐modified Ku70/80 rings, promoting the recruitment of repair proteins like RPA70 and RAD51, thereby enhancing HR repair.

### 2.6 ARN‐3236, a small molecular inhibitor of SIK2, significantly boosts the radiosensitivity of CRC

**FIGURE 6:**
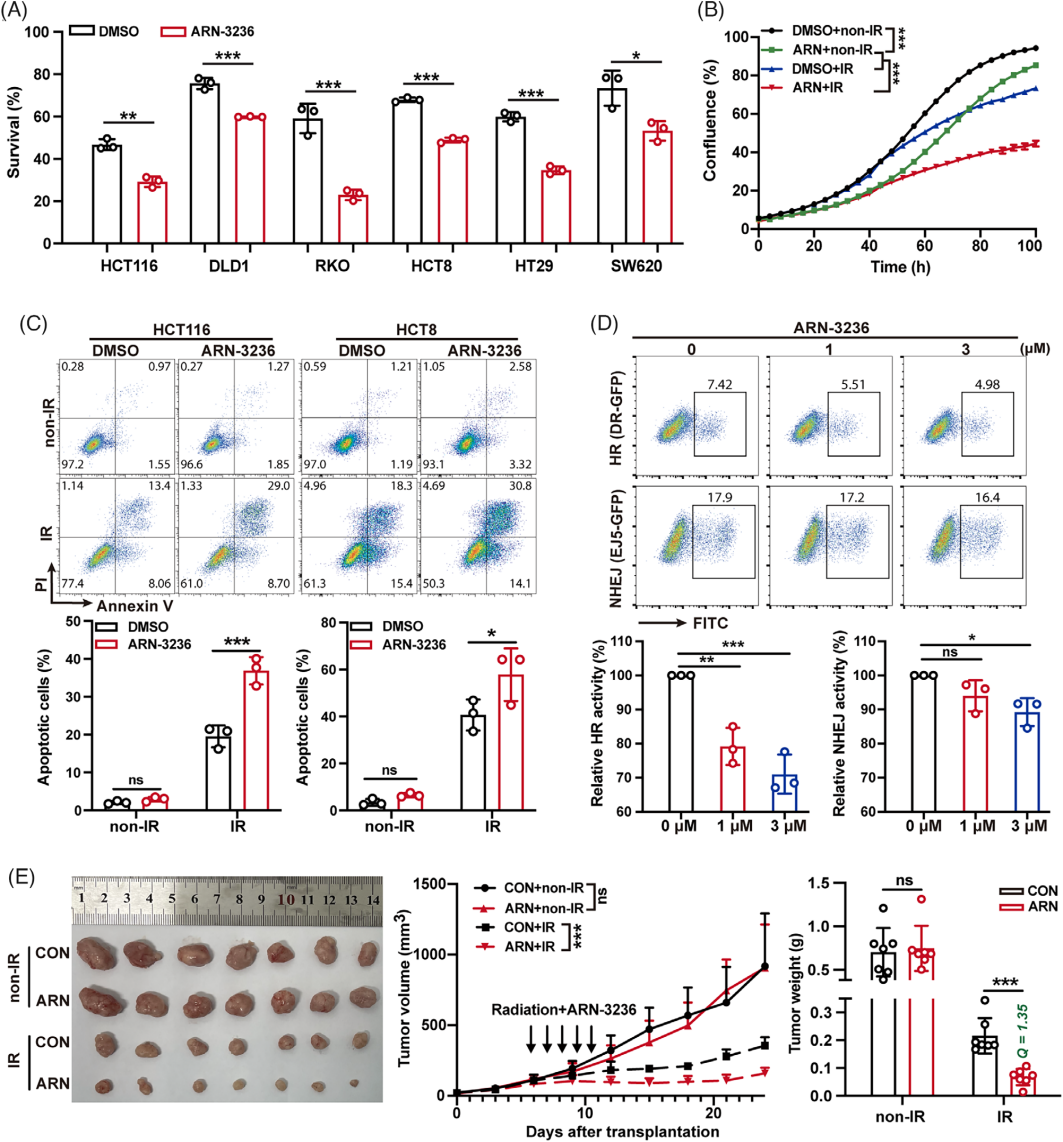
ARN‐3236 significantly boosts the radiosensitivity of colorectal cancer (CRC). (**A)** Colony formation assays were used to assess the effect of ARN‐3236 (5 µM) on cell survival post‐ionizing radiation (post‐IR) in six CRC cell lines. The survival rates post‐IR are represented as mean ± SD of three biological replicates. (**B)** Effects of ARN‐3236 (5 µM) on cell proliferation of HCT8 cells after IR analyzed by the IncuCyte S3 Live‐Cell Analysis System; data are represented as mean ± SEM (*n* = 8). (**C)** Assessment of ARN‐3236 (5 µM) on apoptosis in HCT116 and HCT8 cells 48 h after IR by Annexin V/PI double‐staining assays with flow cytometry (upper, representative pictures of flow cytometry; lower, the percentages of apoptotic cells). Cells treated with DMSO were used as controls; data are represented as mean ± SD of three biological replicates. DMSO, Dimethyl Sulfoxide.(**D)** Direct‐repeat‐green fluorescent protein (DR‐GFP) (HR) and EJ5‐GFP (NHEJ) reporter assays were conducted to assess the effects of ARN‐3236 on DSB repair (upper, representative pictures of flow cytometry; lower, the relative HR or NHEJ activity); data are represented as mean ± SD of three biological replicates. (**E)** Effects of ARN‐3236 on radiotherapy efficacy in the subcutaneous xenograft model. Left, removed xenografts; middle, tumor volume; right, tumor weight; data are represented as mean ± SD (*n* = 7). *Q* value = *E_AB_* /(*E_A_* + *E_B_* − *E_A_* × *E_B_*); *E_A_*, *E_B,_* and *E_AB_* indicated the inhibitory rates of ARN‐3236 (ARN + non‐IR), IR (CON + IR) and ARN‐3236 + IR (ARN + IR), antagonist (*Q* < 0.85), additive (0.85 ≤ *Q* ≤ 1.15), and synergistic (*Q* > 1.15). ARN, ARN‐3236; CON, control, the same volume of solvent as control; non‐IR, without ionizing radiation. ns, not significant; *p < 0.05; **p < 0.01; ***p < 0.001.

In previous studies, we have confirmed that SIK2 mediates the radioresistance of CRC through its kinase activity. We then investigate whether blocking SIK2's kinase activity using inhibitors sensitizes CRC cells to IR. As expected, ARN‐3236, a small molecular inhibitor of SIK2, significantly enhanced the radiosensitivity of multiple CRC cell lines (Figure 6A, Figure S7A). Moreover, ARN‐3236 remarkably increased the growth inhibition and apoptosis effects induced by IR (Figure 6B,C). Consistent with previous mechanistic studies, SIK2 inhibition by ARN‐3236 resulted in a dose‐dependent reduction in HR activity, but only a slight impact on NHEJ activity at higher doses (Figure 6D). Furthermore, inhibition of SIK2 kinase activity with ARN‐3236 significantly reduced IR‐induced VCP hyperphosphorylation (Figure S7B), resulting in abnormal K48‐Ub conjugate accumulation and diminished RPA70 recruitment on chromatin (Figure S7C). Subsequently, we assessed the impact of ARN‐3236 on radiotherapy efficacy in a CRC xenograft model. ARN‐3236 addition increased the tumor inhibition ratio of IR from 69.27% (CON + IR) to 90.41% (ARN + IR), and the combination treatment exhibited a markedly synergistic effect (*Q* = 1.35),^26^, ^27^ although ARN‐3236 alone at administrated dose did not show significant efficacy at the indicated dose (Figure 6E). IHC analysis also revealed that ARN‐3236 resulted in reduced staining of KI67 and increased staining of γH2AX (Figure S7D). Together, these data support the notion that SIK2 inhibitor ARN‐3236 could significantly enhance the sensitivity of radiotherapy both in vitro and in vivo, suggesting its potential as an effective strategy to overcome CRC radioresistance.

## 3 DISCUSSION

Kinases are essential for cellular functions such as DNA repair and cell survival, making them attractive targets for overcoming radioresistance.^10^ This study aimed to identify novel kinases involved in CRC radioresistance and to evaluate if their inhibitors could serve as radiosensitizers. We utilized kinase‐targeted CRISPR‐Cas9 screening combined with next‐generation sequencing to identify CRC radioresistance kinases. Our results pinpointed *SIK2, DYRK4, CAMK4*, and *HIPK4* as candidate radioresistant kinases in CRC. We selected *SIK2* as our primary research target because it demonstrated the strongest radioresistance function among the candidates. Importantly, *SIK2* has a specific small‐molecule inhibitor, ARN‐3236, which can be used as a potential radiosensitizer. Future studies will further investigate the roles and specific molecular mechanisms of *DYRK4*, *CAMK4*, and *HIPK4* in CRC radioresistance. Additionally, the enrichment of known radioresistance kinases in our screening, such as *CLK2*,^15^ *CALM2*,^16^ *ATM*,^8^ and *COASY*,^28^ validates the reliability of our screening approach.

Salt‐inducible kinases (SIKs)—including SIK1, SIK2, and SIK3—belong to the AMP‐activated protein kinase (AMPK) family and are serine/threonine kinases. These isoforms are similar in three key domains.^29^ SIK1 is located on chromosome 21, while SIK2 and SIK3 are on chromosome 11. SIK1 is predominantly expressed in the adrenal cortex, as well as in the adipose and neural tissues. In contrast, SIK2 and SIK3, while ubiquitous, show abundant expression in adipose and neural tissues, respectively.^30^ Our real‐time PCR (RT‐PCR) analysis revealed low mRNA levels of SIK1 in CRC cells, while the mRNA levels of SIK2 and SIK3 were relatively high, indicating that SIK2 and SIK3 are mainly expressed in CRC cells (Figure S8A).

SIKs are crucial for various metabolic processes, but their roles in cancer are complex and not fully understood.^30^ Despite their structural similarities, the SIK family members have distinct biological functions. SIK1 predominantly acts as a tumor suppressor by inhibiting epithelial‐mesenchymal transition and regulating gene expression to limit tumor progression. Conversely, SIK3 demonstrates a dual role: it promotes cell proliferation in breast and ovarian cancers by facilitating the G1/S transition, but inhibits proliferation in non‐small cell lung cancer (NSCLC) through modulation of AP1 and IL6 signaling. Compared with SIK1 and SIK3,^30^ the research on the oncogenic role of SIK2 in tumors is more extensive,^31^, ^32^, ^33^ especially in ovarian and breast cancers.^12^, ^13^, ^34^, ^35^, ^36^, ^37^ SIK2 phosphorylates a range of substrates contributing to ovarian cancer cell motility, migration, metastasis, and the maintenance of breast cancer stemness.^34^, ^35^, ^36^, ^37^, ^38^ However, SIK2's function in CRC remains largely unexplored. Our research uncovers its novel function in conferring radioresistance in CRC. Interestingly, our screening revealed that SIK1 was not enriched and SIK3 was ranked low—288th in the 5 Gy group and 584th in the 10 Gy group. Unexpectedly, the pan‐SIK inhibitor^39^ HG‐9‐91‐01, which targets all SIK proteins, did not sensitize CRC cells to IR. Instead, it increased cell survival and reduced IR‐induced apoptosis (Figure S8B–D). Considering HG‐9‐91‐01 can target all the SIK proteins^39^ while ARN‐3236 has minimal inhibitory effects on SIK1 and SIK3, as well as other members of the AMP‐activated protein kinase (AMPK) family,^14^ the results suggested that inhibiting SIK1 and SIK3 might counteract the radiosensitizing effect of SIK2 inhibition, leading to the observed non‐sensitizing effect of HG‐9‐91‐01. These findings collectively indicate that SIK2 is the primary regulator of CRC radioresistance within the SIK family.

DSB repair pathways, including error‐free HR and error‐prone NHEJ, are crucial for understanding radiation resistance in tumor cells.^5^, ^6^ Our study found that SIK2 knockdown or inhibition with ARN‐3236 significantly reduced HR activity while leaving NHEJ largely unaffected. Additionally, although SIK2 has been reported to promote centrosome separation in ovarian cancer cells by phosphorylating C‐Nap1,^38^ our study found no notable cell cycle distribution changes in non‐irradiated SIK2 knockdown CRC cells compared to controls. However, post‐irradiation, the knockdown group exclusively exhibited sustained G2/M phase arrest (Figure S3C). HR, which uses the sister chromatid as a homologous sequence donor, is mainly active during the S and G2 phases of the cell cycle.^7^ Upon DNA damage, cells typically arrest the cell cycle to allow time for repair. However, when HR repair is impaired, G2/M phase arrest is prolonged due to unresolved DNA damage. This is consistent with our findings, where SIK2 knockdown attenuated HR repair and significantly prolonged G2/M phase arrest after IR.

A previous study reported that SIK2 could sensitize PARP inhibitors for its inhibition repressed by the expression of DNA repair genes, such as FANCD2, EXO1, and XRCC4 in ovarian and triple‐negative breast cancers.^12^ Furthermore, SIK2 has been identified as a synthetic lethal target of FANCA and a sensitizer to paclitaxel, due to its role in spindle assembly and centrosome separation.^13^, ^40^ However, established mechanisms do not explain the significant inhibitory effect of SIK2 on HR repair observed in our study. Here, we discovered that SIK2 enhances VCP's ability to remove chromatin K48–Ub conjugates through VCP hyperphosphorylation, thereby increasing HR repair efficiency. This finding was further supported by point mutation recovery experiments, which demonstrated that hyperphosphorylation of VCP at serines 770 and 784 is closely linked to its function of extracting K48–Ub conjugates. Additionally, it was previously reported that SIK2 co‐localizes with VCP at the ER membrane, enhancing VCP ATPase activity and ERAD function via Ser‐770 phosphorylation.^24^ Our research extends this understanding by demonstrating that SIK2 also influences the extraction of chromatin‐bound proteins by VCP, thereby promoting HR repair and expanding our knowledge of SIK2's regulatory role on VCP.

VCP is involved in DSB repair by extracting K48–Ub conjugates from chromatin,^19^, ^22^, ^23^, ^41^ with key substrates including L3MBTL1^23^ and Ku.^22^ VCP promotes 53BP1 recruitment by removing L3MBTL1 from DNA damage sites.^23^ Our study showed that SIK2 knockdown reduced VCP extraction activity, leading to the abnormal accumulation of K48–Ub conjugates and Ku on chromatin in the late stage of DNA damage response. Interestingly, 53BP1 enrichment was not significantly impacted, possibly due to its early recruitment to damage sites post‐irradiation,^23^ which occurs within minutes and was not captured by our 6‐h post‐irradiation protein detection timeline. In our HR/NHEJ report assay, VCP knockdown notably decreased HR repair efficiency consistently across different siRNAs (Figure 4D), aligning with previous studies.^22^ However, its effect on NHEJ repair efficiency was variable: two siRNAs increased NHEJ efficiency upon knockdown, while one showed a decrease (data not shown). Overall, VCP knockdown predominantly reduced HR rather than NHEJ efficiency. Although 53BP1 can promote NHEJ by antagonizing BRCA1, the rapid‐kinetic NHEJ, which repairs most DSBs, functions independently of 53BP1. Furthermore, the immediate‐early loading of Ku70/80 guides easily repairable DSBs toward this highly efficient rapid‐kinetic NHEJ.^42^ VCP not only removes Ku70/80 post‐repair but also extracts Ku from unrepaired DNA ends pre‐ligation, influencing pathway selection toward HR over NHEJ.^22^ Consequently, the decrease in HR efficiency due to abnormal Ku accumulation is predominant, corroborated by our findings of reduced RPA and RAD51 recruitment in HR repair.

**FIGURE 7:**
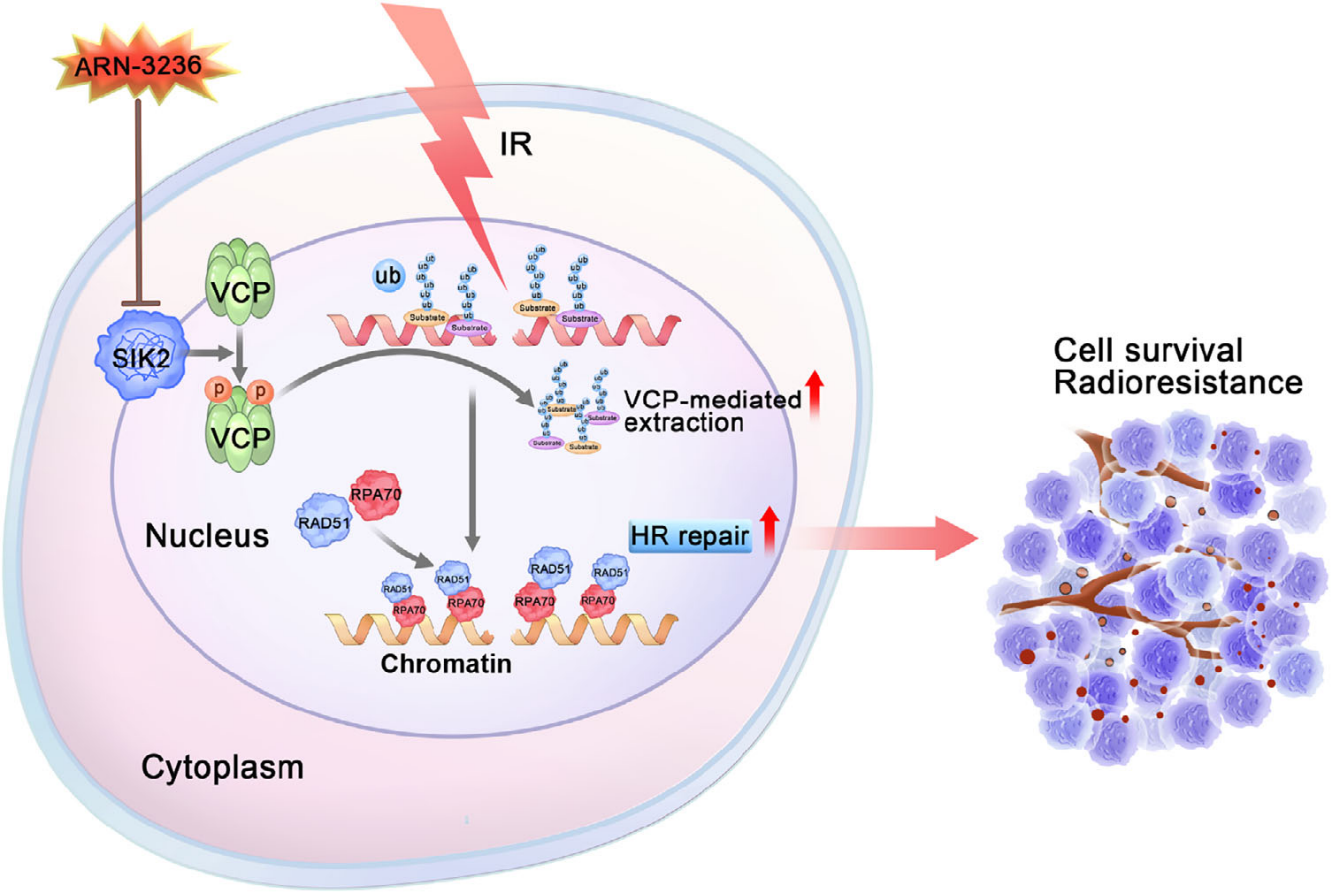
The schematic illustrates how SIK2 confers resistance to ionizing radiation (IR) in colorectal cancer.

In conclusion, our research uncovers a novel mechanism by which SIK2 contributes to the radioresistance of CRC: SIK2 binds to VCP and promotes its hyperphosphorylation, which enhances VCP's function in extracting K48‐linked ubiquitin‐conjugated proteins, such as K48‐modified Ku70/80 rings, from chromatin. This process facilitates the recruitment of RPA and RAD51 to DNA damage sites, thereby strengthening HR‐mediated DNA repair and contributing to CRC radioresistance (Figure 7). Importantly, ARN‐3236, an oral SIK2 inhibitor, significantly increases the radiosensitivity of CRC cells, both in vitro and in vivo, emerging as a potential radiosensitizer in CRC treatment (Figure 7).

## 4 LIMITATIONS

Our study has several limitations. While our findings demonstrated that the inhibitor ARN‐3236 significantly enhances radiosensitivity in CRC cells, its clinical potential needs further validation, such as evaluation in organoid models. Additionally, although we validated the clinical relevance of SIK2 using patient data from public databases, future studies could further reinforce these findings by collecting and analyzing biopsy specimens from LARC patients receiving neoadjuvant radiotherapy.

## 5 MATERIALS AND METHODS

### 5.1 RNA interference, plasmids, and transfections

Negative control (siNC) and specific siRNAs (Table S4) were synthesized by TSINGKE. Human SIK2 and VCP cDNA were amplified from pCDH‐SIK2 (NO. OENM_015191) and pENTER‐VCP (NO. CH897720) plasmids purchased from WZ Biosciences. Human SIK2^K49M^, VCP^S784A^, VCP^S784D^, VCP^S770A^, and VCP^S770D^ cDNAs were introduced using Mut Express II Fast Mutagenesis Kit V2 (No. C214‐01/02, Vazyme). The sequences of primers in point mutation are shown in Table S5. VCP‐DD (S770D S784D) and VCP‐AA (S770A S784A) mutants were introduced based on VCP^S784A/D^ cDNAs using VCP^S770A/D^ sense and antisense primers. SIK2, SIK2^K49M^, VCP, VCP‐DD, and VCP‐AA cDNAs were amplified and inserted into the pcDNA4.0 vector to construct transient overexpressing plasmid with a Myc tag. All transfections were performed using Lipofectamine 3000 (No. L3000008, Invitrogen) according to the manufacturer's instructions.

### 5.2 Generation of stable cell lines

Human SIK2, SIK2^K49M^, VCP, VCP‐DD, and VCP‐AA cDNAs were cloned into the pCDH‐EF1‐MCS‐T2A‐Puro plasmid (System Bioscience). SIK2, SIK2^K49M^, and VCP cDNAs were also cloned with an N‐terminal 3×Flag tag into the same plasmid. shRNAs (Table S6) were inserted into the pLKO.1(Sigma‐Aldrich) or pLKO‐Tet‐On (Novartis) vector. These plasmids were cotransfected into 293T cells along with psPAX2 and pMD2.G lentiviral packaging plasmids to produce recombinant lentiviruses. Stable gene overexpression or knockdown in CRC cells was achieved through lentiviral transduction followed by puromycin selection.

### 5.3 Kinase‐targeted CRISPR‐Cas9 screen

The screen was performed using the Protein kinase‐Dharmacon Edit‐R Lentiviral sgRNA Pooled Screening Library, targeting 697 protein kinases, according to the manufacturer's guidelines. sgRNA copy numbers from X‐ray‐treated and ‐untreated groups were compared, and MAGeCK‐negative selection was used to identify potential radioresistance genes.^43^ Knockdown of these genes increases radiosensitivity, making cells with targeted sgRNAs more prone to X‐ray‐induced death, leading to a reduction in sgRNA copy numbers in surviving cells. Radioresistance kinase candidates were defined by two criteria: (i) a MAGeCK‐negative rank within the top 100 and (ii) at least two downregulated sgRNAs with copy numbers exceeding 50 (Figure 1A).

### 5.4 Colony formation assay

Cells were seeded into six‐well plates at a concentration of 1000–1500 cells per well. After 24 h, cells were treated with indicated doses of X‐rays (0, 2, 4, or 6 Gy) and then cultured for 9–14 days. In experiments related to ARN‐3236 or HG‐9‐91‐01, cells were treated with ARN‐3236, HG‐9‐91‐01, or Dimethyl Sulfoxide (DMSO) at the indicated doses for 24 h and then subjected to X‐ray treatment. ARN‐3236, HG‐9‐91‐01, or DMSO in the culture medium was replaced with a fresh medium 24 h after IR, and cells were further incubated for 9–14 days. Colonies were fixed with fixation solution (methanol: acetic acid = 5:1) for 20 min at room temperature and then stained with a solution of 0.5% crystal violet in methanol for 2 h. Colonies containing over 50 cells were counted.

### 5.5 Reverse transcription and real‐time PCR

Total RNA was extracted from cells with TRIzol Reagent (No. 15596026, Invitrogen), according to the manufacturer's protocol, and quantified with Nanodrop 2000 (Thermo Fisher Scientific). Total RNA was reverse transcribed into cDNA using the HiScript Reverse Transcriptase Kit (No. R123‐01, Vazyme). Gene expression levels were assessed using RT‐PCR with ChamQ SYBR qPCR Master Mix (No. Q311‐02, Vazyme) and specific primers (Table S7). GAPDH was used as the internal control.

### 5.6 Nude mouse xenograft model and radiotherapy

The animal experiments were authorized by the Sun Yat‐sen University Cancer Center Animal Care and Use Committee and adhered to all relevant ethical guidelines for animal research (Approval number: L025501202206013). Female BALB/c nude mice (4‐5 weeks, 15–18 g) were acquired from Gempharmatech‐GD.

To assess the effect of radiotherapy on SIK2 expression in vivo, 5 × 10^6^ HT29 cells stably overexpressing mCherry protein were injected subcutaneously into the mice's flank. One week later, the mice received 1 Gy/day X‐ray treatment for ten consecutive days. After irradiation, xenograft growth was tracked, and the tumor was collected when the growth rate was significantly accelerated. We extracted primary cells from tumor tissues and selected the tumor cells that express mCherry protein by flow cytometry (Figure S1C).

To establish subcutaneous xenografts with DOX‐induced SIK2 knockdown, 3 × 10^6^ HCT116 cells harboring a Tet‐On inducible SIK2 knockdown construct were suspended in 100 µL PBS and injected subcutaneously into the mice's flanks (*n* = 6). Four days post‐injection, doxycycline (2000 ppm, No. C11300‐2000, Research Diets) was administered via feed to trigger gene knockdown. Three days later, the mice underwent X‐ray treatment at 2 Gy/day for seven consecutive days, protecting non‐xenograft areas with a lead shield.

In experiments involving ARN‐3236, xenograft models were generated by subcutaneously transplanting approximately 5 mm^3^ tumor sections from CRC cell xenografts into the flanks (*n* = 7). One week after transplantation, mice were treated selectively with ARN‐3236 and X‐rays. ARN‐3236 was orally administered at 40 mg/kg for 7 days. The same volume of solvent was administered by gavage as control. The pattern of radiotherapy was 2 Gy/day for the first three days, followed by 1 Gy/day for 4 days, totaling 10 Gy. The combined group (ARN + IR) received the same ARN‐3236 dosage and was subjected to irradiation 4 h post‐oral administration, following the same radiotherapy pattern.

Xenograft growth was monitored, and tumor volumes were calculated using the formula: 0.52 × width^2^ × length.^43^ Two weeks after radiotherapy, the mice were euthanized, and the tumors were excised for weight measurement and following pathological analysis

### 5.7 Western blot

Cells were lysed with lysis buffer (Beyotime) containing protease and phosphatase inhibitors (TOPSCIENCE). Protein concentrations were measured using a BCA Protein Assay Kit (KeyGen Biotech). Proteins were analyzed by sodium dodecyl sulfate‐polyacrylamide gel electrophoresis (SDS‐PAGE) for general detection. Phosphorylated proteins were specifically detected using SuperSep Phos‐tag polyacrylamide gel electrophoresis, which captures phosphorylated proteins during SDS‐PAGE, allowing their differentiation from non‐phosphorylated proteins due to the Phos‐tag's specific binding to phosphate groups.^21^ Protein samples were separated by SDS‐PAGE or Phos‐tag SDS‐PAGE (FUJIFILM Wako) and transferred onto a polyvinylidene difluoride membrane (Roche), which was blocked with 5% milk in TBST (50 mM Tris‐HCl, 150 mM NaCl, 0.5% Tween‐20, pH 7.4) and incubated overnight at 4°C with specific antibodies (Table S8). Subsequently, the membrane was incubated with horseradish peroxidase (HRP)‐conjugated goat anti‐rabbit IgG (1:3000; No. 7074; Cell Signaling Technology) or anti‐mouse IgG (1:3000; No. 7076; Cell Signaling Technology). Signals were visualized using an ECL Plus chemiluminescence reagent kit (4A Biotech).

### 5.8 Immunohistochemistry assay

Following a previously described methodology,^44^, ^45^ IHC assays were performed to assess protein levels in xenograft tissue samples. Formalin‐fixed, paraffin‐embedded tissue sections of 4 mm thickness were subjected to deparaffinization, rehydration, antigen retrieval, and inactivation of endogenous peroxidase. Post‐blocking, the sections were incubated overnight at 4°C with primary antibodies (Table S8). This was followed by incubation with an HRP‐conjugated secondary antibody, visualization using an Envision Detection Kit (Dako), and counterstaining with hematoxylin.

### 5.9 Immunofluorescence

Cells under different treatments in confocal dishes were fixed with 4% paraformaldehyde (No. BL539A, Biosharp), permeabilized with 0.5% Triton X‐100 (No. V900502, Sigma‐Aldrich), and blocked with 3% BSA (Beyotime). The cells were then incubated with indicated primary antibodies (Table S8) at 4°C overnight, followed by incubation with goat anti‐mouse IgG secondary antibody, Alexa Fluor Plus 488 (1:200; No. A32723; ThermoFisher) or goat anti‐rabbit IgG secondary antibody, Alexa Fluor 594 (1:200; No. A11012; ThermoFisher) for 1 h at room temperature in the dark. The samples were costained with DAPI (Beyotime) for 5 min and examined by confocal laser scanning microscopy.

### 5.10 Analysis of the efficiency of HR and NHEJ

We performed the DR‐GFP and EJ5‐GFP reporter assays to quantify the activities of HR and NHEJ. The reporter assays allow for measurement repair via HR or NHEJ of a DSB produced by the endonuclease I‐SceI in GFP‐positive cells.^18^ The U2OS DR‐GFP and EJ5‐GFP reporter cell lines were gifts from Dr. Mu‐Yan Cai, Sun Yat‐sen University Cancer Center (SYSUCC). Cell transfected with siRNA or treated with ARN‐3236, followed by infection with I‐SceI adenovirus 24 h later. The activity of HR and NHEJ was measured by flow cytometric quantification of viable GFP^+^ cells 48 h after I‐SceI adenovirus infection.

### 5.11 Statistical analysis

Statistical analyses were conducted using SPSS 20.0 (Chicago), GraphPad Prism 9, and R software (version 4.1.0). Comparisons between two groups were performed with a two‐tailed Student's *t*‐test. For comparisons involving three or more groups, analysis of variance was utilized. Chi‐square tests were applied to evaluate differences in proportions among groups. Kaplan–Meier curves were generated to assess OS, with differences in survival rates evaluated using the log‐rank test. Statistical significance was defined as a *p* value of less than 0.05.

## AUTHOR CONTRIBUTIONS

*Study concept and design*: Ran‐yi Liu and Yuan Meng. *In vitro functional studies and data analysis*: Yuan Meng, Shuo Li, Da‐shan Lu, Xue Chen, and Gao‐yuan Wang. *Animal experiments and data analysis*: Yuan Meng, Da‐shan Lu, Lu Li, and You‐fa Duan. *Mechanistic studies and data analysis*: Yuan Meng, Shuo Li, and Xue Chen. *Statistical analysis*: Yuan Meng and Shuo Li. *Analysis and interpretation of data and writing of the manuscript*: Ran‐yi Liu and Yuan Meng. All authors have read and approved the final manuscript.

## CONFLICT OF INTEREST STATEMENT

Author Wenlin Huang is an employee of Guangzhou DoublleBioproduct Co., Ltd., but has no potential relevant financial or non‐financial interests to disclose. The other authors have no conflicts of interest.

## ETHICS STATEMENT

The animal experiments were authorized by the Sun Yat‐sen University Cancer Center Animal Care and Use Committee and adhered to all relevant ethical guidelines for animal research (Approval number: L025501202206013). Since clinical data and gene expression profiles of CRC patients were obtained from the Gene Expression Omnibus (GEO) databases, no further ethical approval is needed for this study.

## Supporting information



Supporting Information

## Data Availability

The authenticity of this article was validated by uploading the raw data to the Research Data Deposit public platform (www.researchdata.org.cn) with approval number RDDB2024890937.
