# Supplementary material for: Salt‐inducible kinase 2 confers radioresistance in colorectal cancer by facilitating homologous recombination repair
Source: MedComm (2020). 2025 Jan 28;6(2):e70083. doi: 10.1002/mco2.70083 (PMC11774237; doi:10.1002/mco2.70083)
Supplement: Supplementary file 1 — Supporting Information [file MCO2-6-e70083-s001.pdf]

Supplementary Materials

Salt-inducible kinase 2 confers radioresistance in colorectal cancer by facilitating homologous recombination repair

Yuan Meng[1,#], Shuo Li[1,2,#], Da-shan Lu[1,#], Xue Chen[1], Lu Li[1], You-fa Duan[1], Gao-yuan Wang[1], Wenlin Huang[1,3], Ran-yi Liu[1,*].

[1] State Key Laboratory of Oncology in South China, Guangdong Provincial Clinical Research Center for Cancer, Sun Yat-sen University Cancer Center, Guangzhou 510060, China.

[2] Department of Pathology, Sun Yat-sen University Cancer Center, Guangzhou 510060, China.

[3] Guangdong Provincial Key Laboratory of Tumor Targeted Drugs & Guangzhou Enterprise Key Laboratory of Gene Medicine, Guangzhou DoublleBioproduct Co., Ltd., Guangzhou 510535, China

[#] Yuan Meng, Shuo Li and Da-shan Lu contributed equally to this study.

* **Correspondence:** Ran-yi Liu, State Key Laboratory of Oncology in South China, Guangdong Provincial Clinical Research Center for Cancer, Sun Yat-sen University Cancer Center, Guangzhou, 510060, China. E-mail: liury@sysucc.org.cn

Table of Contents

Section	Page
**Supplementary Methods**	**3-8**
**Supplementary Figures**	**9-20**
**Figure S1.** The screening of candidate radioresistant genes.	**9**
**Figure S2.** SIK2 increases the radioresistance of CRC cells.	**10-11**
**Figure S3.** SIK2 promotes DNA damage repair in colorectal cancer.	**12-13**
**Figure S4.** The heatmap presentation of DNA damage repair gene expression**.**	**14-15**
**Figure S5.** SIK2 promotes the radioresistance of CRC cells by interacting with VCP.	**16**
**Figure S6.**	**17**
**Figure S7.** ARN-3236 significantly sensitizes CRC cells to radiation both *in vitro* and *in vivo*.	**18-19**
**Figure S8.** SIK2 plays a specific role in promoting radioresistance among the SIK family.	**20**
**Supplementary Tables**.	**21-28**
**Table S1.** Candidate radioresistance genes selected in cells treated with 10Gy X-ray by MAGeCK analysis.	**21-22**
**Table S2.** Candidate radioresistance genes selected in cells treated with 5Gy X-ray by MAGeCK analysis.	**23-24**
**Table S3.** Detailed information about the sensitivity enhancement ratio of SIK2 knockdown in different CRC cells.	**25**
**Table S4.** The sequences of siRNAs.	**26**
**Table S5.** The sequences of primers in point mutation.	**26**
**Table S6.** The information of shRNAs.	**27**
**Table S7.** The sequences of real-time PCR Primers	**27**
**Table S8.** The information of primary antibodies.	**28**

Supplementary Methods

Cell culture and reagents

The CRC cell lines HCT116, HCT8, RKO, SW620, SW480, DLD1, and HT29, as well as the HEK293T cell line, were sourced from the American Type Culture Collection (Manassas, VA, USA) and cultured according to standard protocols. All cell lines underwent short tandem repeat (STR) authentication, and have been confirmed mycoplasma-free using the PCR Detection Kit (NO. D101-02, Vazyme).

Methods of drug formulation

The compounds ARN-3236 (NO. T5993) and HG-9-91-01 (NO. T4599) were obtained from TOPSCIENCE (Shanghai, China). In cellular experiments, ANR-3236 and HG-9-91-01 are prepared as a 10 mM stock solution using dimethyl sulfoxide (DMSO) dissolution. The stock solution can be diluted to different working concentrations as required during the experiment. In animal experiments, the dissolution system for ARN-3236 was: 100% Ethanol, 5%; PEG-300 (Sigma, 202371), 30%; 20% tween 80 (Sigma, P5188), 10%; and ddH2O, filling up the remaining volume. After adding PEG-300, we used a Qsonica Q125 sonicator to homogenize the mixture. The frequency is 20 kHz, 100% amplitude for 10 seconds. In between sonications, the tube will be put on ice for at least 30 seconds to prevent too much heating. Sonicate the mixture until there are no longer visible particles floating in the solution.

Clinical Information Analysis

Clinical data and gene expression profiles of CRC patients were obtained from the (Gene Expression Omnibus) GEO databases, specifically from datasets GSE133057

and GSE17536. The cutoff value for SIK2 expression was determined based on OS using ROC curve. Patients were subsequently categorized into high or low SIK2 expression groups according to this value. Tumor response to nCRT was evaluated using tumor regression grade (TRG), which was proposed in the 8th Edition of the American Joint Committee on Cancer (AJCC) Staging Manual System. TRG 0 was considered as a complete response (CR) to nCRT, while TRG 1-3 was considered as non-CR. The distribution of SIK2 expression levels between patients with complete (CR) and incomplete (non-CR) responses to nCRT was analyzed. Kaplan–Meier curves were generated to assess overall survival.

Calculation of sensitivity enhancement ratio

The sensitivity enhancement ratio (SER) is commonly used to evaluate the sensitivity of cells to irradiation under different conditions. We calculated the SER of the SIK2 knockdown group relative to the control group using the survival of CRC cells at different radiation doses in colony formation assays. It has been demonstrated that cell death after receiving different doses of irradiation usually follows a linear-quadratic model[1, 2]. We entered the survival rates of different groups at different radiation doses of irradiation in GraphPad Prism software and fitted the survival curve model using the Linear quadratic model. The radiation dose required to kill 63% of the cells (D_0) was calculated using the model. D_0 is also known as the mean lethal dose. The SER of the knockdown group was then calculated by dividing the D_0 of the control group by the D_0 of the knockdown group. An SER greater than 1 indicates that the cells in that group are more sensitive to irradiation.

Cell proliferation assay

HCT116 and HCT8 cells varying in SIK2 expression were seeded in a 96-well plate at an initial 2500 cells per well, after 24 hours, cells were treated with different doses of X-rays (HCT116: 0 or 4 Gy; HCT8: 0 or 8 Gy). HCT8 cells were seeded into 96-well plates at a concentration of 2000 cells per well. Cells were treated with ARN-3236 or DMSO for 24 hours and then subjected to X-ray treatment. ARN-3236 or DMSO in the culture medium was replaced with a fresh medium 24 hours after IR. All the plates with different treatments were placed in an IncuCyte S3 Live-Cell Analysis System, where real-time images were captured every 4 h for 100 h. Photographs of the cells were taken from four separate regions of each well using a 10 × objective. Cell proliferation was evaluated by the degree of cell confluence.

Apoptosis assay

HCT116 and HCT8 cells with stable SIK2 knockdown were seeded into 6-well plates at 2 $\times 10^5$ cells/well. After 24 h attachment, cells were exposed to X-rays (0 or 6 Gy) and cultured for 48 h. HCT116 and HCT8 cells were seeded into 6-well plates at 2 $\times 10^5$ cells/well, treated with ARN-3236, HG-9-91-01, or DMSO for 24 h, and exposed to IR. Replaced ARN-3236, HG-9-91-01, or DMSO with fresh culture medium, and harvested cells after 48 h. Apoptosis was detected by the Annexin V/propidium iodide (PI) double-staining kit (NO. FXP023-100, 4A Biotech). The apoptosis rates were measured and analyzed by flow cytometry and FlowJo software.

Comet assay

Cells were collected via trypsinization 2 h post-IR (18 Gy) for comet assay. This analysis used the CometAssay Kit (NO. 4250-050-K, R&D Systems), which was applied according to the manufacturer's protocol. Comet images were captured using a fluorescence microscope, and the DNA percentage in comet tails was quantitatively assessed using OpenComet software.

Cell cycle assay

Cells were collected at indicated time points after irradiation and fixed in 95% ethanol for 24 hours at 4 °C. After fixation, the resuspended cells were washed with PBS precooled by ice and incubated with propidium iodide (PI) solution containing RNase (NO. FXP021-200, 4A Biotech) for 30 min at 37 °C water bath in the dark. The stage of the cell cycle was detected by flow cytometry and analyzed by FlowJo software.

mRNA-sequencing and analysis

Poly(A) containing mRNA sequencing was performed by Mingma Technologies (Shanghai, China). Poly(A) containing mRNA was extracted from total RNA using magnetic beads with oligo-dT and then fragmented randomly. The cleaved RNA fragments were then reverse transcribed into first-strand cDNA by reverse transcriptase using random hexamer primers for RT priming and reverse transcription, followed by second-strand cDNA synthesis using DNA polymerase I and RNase H. The reverse transcription product is subjected to terminal repair, followed by adding an A base at the 3' end. Subsequently, the fragments were ligated with an Illumina universal adapter. After PCR amplified, the ligation products were purified to remove the

incompletely ligated fragment and the self-ligated adapter. Library sizes were detected using an Agilent 2100 Bioanalyzer and library concentrations were detected using Qubit 2.0. The DNA sequencing was performed according to Illumina's standard protocol. Read quality was calculated for all samples by using FastQC (version 0.11.9) software with the default parameters. STAR (version 2.7.8a) and RSEM (version 1.3.3) software were used to align RNA sequences to gene regions and accurately quantify gene or transcript expression levels from RNA-seq data. Then edgeR (version 3.39.6) package in R (version 4.2.1.) was used to perform TMM (trimmed mean of M values) and CPM (count per million) normalization for the gene expression. TMM is mainly used to normalize the depth of sequencing in different samples and estimate the expression level for each sample. CPM is used to normalize the amount of read count expression between samples. TMM and CPM normalized read counts of genes were applied to compare between two samples in the downstream analysis.

The Human Gene Set: M14832 (https://www.gsea-msigdb.org/gsea/msigdb/human/geneset/GOBP_SIGNAL_TRANSDUCTION_IN_RESPONSE_TO_DNA_DAMAGE.html) was used to retrieve genes related to DNA damage repair. Heatmap plots of selected genes showing their variation among different samples were generated by ggplot2 (version 3.3.6) in R, version 4.2.1.

blot.

Co-immunoprecipitation assay

Co-immunoprecipitation (Co-IP) assays were performed with the Anti-Flag Magnetic Beads (NO. HY-K0207, MCE) according to the manufacturer’s instructions. In brief,

the total cell lysates and the beads were incubated overnight at 4 °C with shaking. Followed by five times washing with TBST (50 mM Tris-HCl, 150 mM NaCl, 0.5% Tween-20, pH 7.4), the beads were boiled at 100 °C for 5 min with the 1× protein loading buffer, and the supernatant was collected for next WB analysis.

Subcellular fractionation

Subcellular fractionation was isolated using a subcellular fractionation kit (NO. 78840, ThermoFisher), following the manufacturer's protocol. The protein lysates from each fraction were subsequently analyzed through Western Blot.

Reference

1. Bodgi L, Canet A, Pujo-Menjouet L, Lesne A, Victor JM, Foray N. Mathematical models of radiation action on living cells: From the target theory to the modern approaches. A historical and critical review. *J Theor Biol.* Apr 7 2016;394:93-101.

2. Brenner DJ. The linear-quadratic model is an appropriate methodology for determining isoeffective doses at large doses per fraction. *Semin Radiat Oncol.* Oct 2008;18(4):234-239.

Figure S1

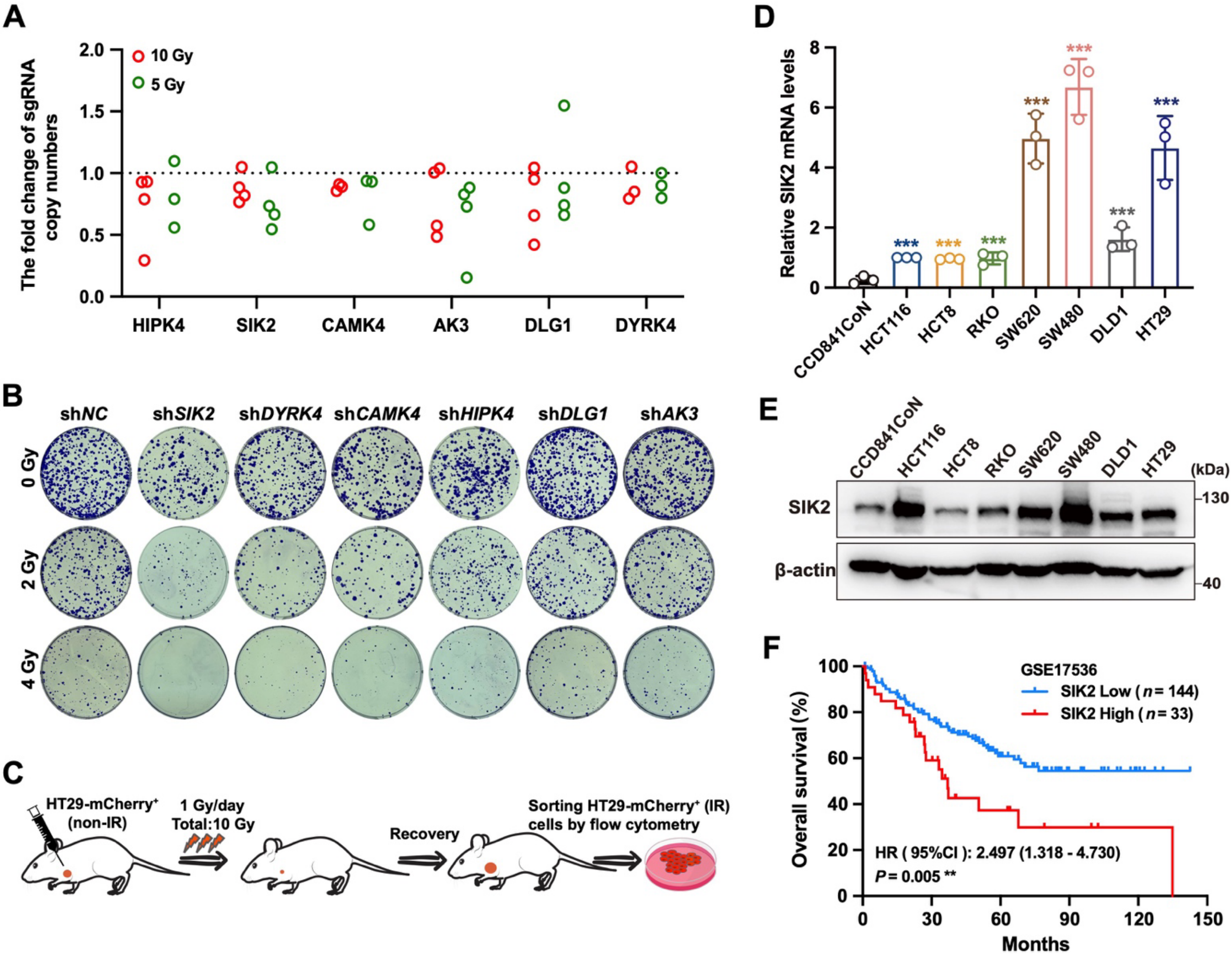

Figure S1. The screening of candidate radioresistant genes. A, The fold change of sgRNA copy numbers targeting 6 candidate radioresistant genes in HCT116 cells (10 Gy or 5 Gy VS 0 Gy). **B**, The representative colony formation pictures of HCT116 cells with stable knockdown of indicated genes after IR. shNC, negative control. **C**, The schematic diagram depicts the procedure to generate HT29-mCherry$^+$ cells derived from xenografts with ionizing radiation (IR) and without ionizing radiation (non-IR). **D** and **E**, Relative mRNA level (**D**) and protein level (**E**) of SIK2 in the normal intestinal epithelial and CRC cells. CCD841CoN was used as control, data are represented as mean ± SD of three biological replicates; statistical significance symbols in different colors, compared to the control. **F**, Overall survival curves according to high or low SIK2 expression levels. Analysis of a published CRC cohort (GSE17536). The cut-off value for SIK2 level was deduced according to OS using an ROC curve, and patients were categorized into two groups based on SIK2 level (high or low). ***P* < 0.01; ****P* < 0.001

Figure S2

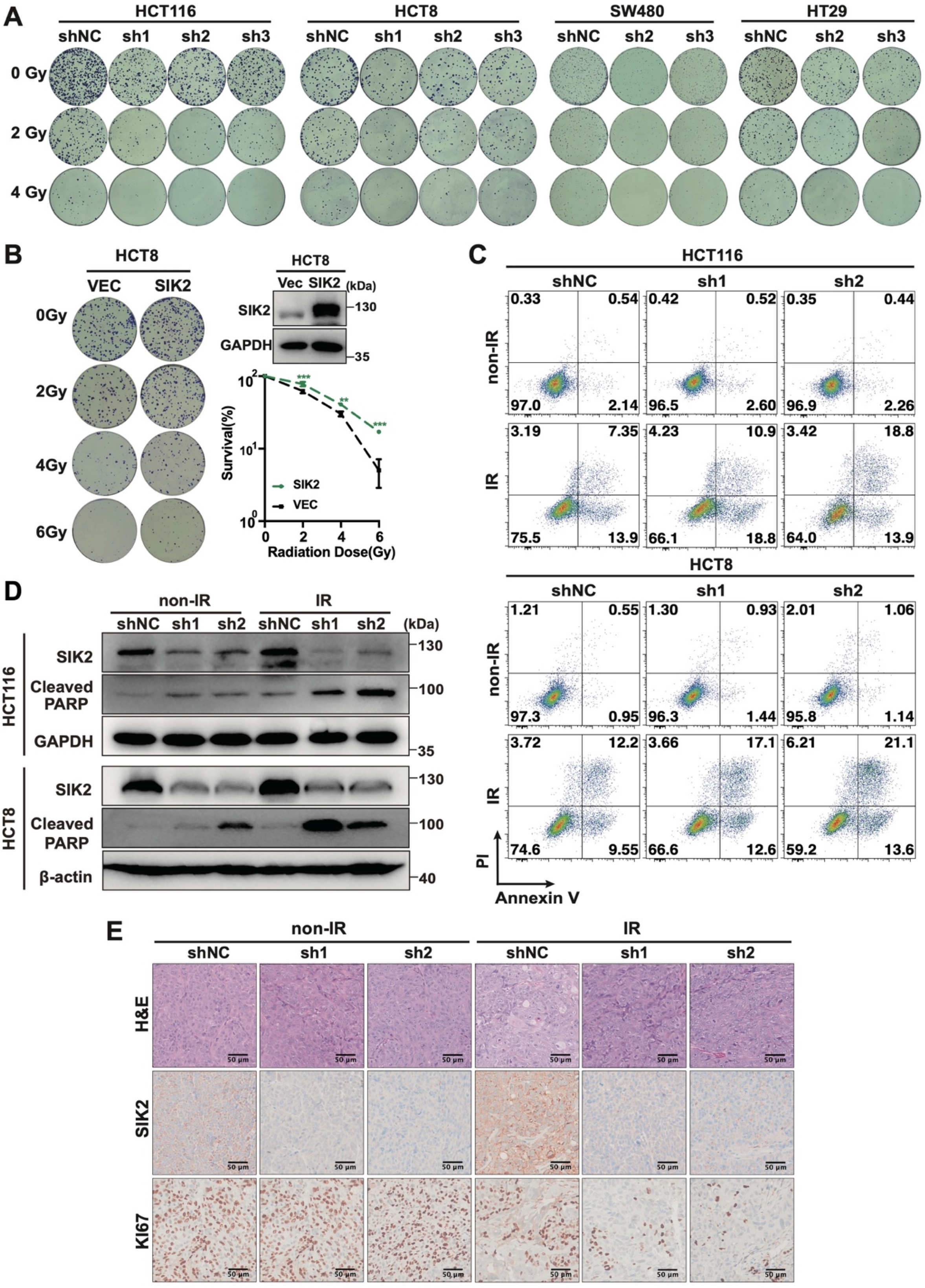

Figure S2. SIK2 increases the radioresistance of CRC cells. A, Representative colony formation pictures of Figure. 2A-D. **B,** Colony formation assays were used to assess survival

after IR for HCT8 cells with SIK2 overexpression. Left: representative colony formation pictures, right: WB pictures and survival rate. Vec, lentiviral vector; SIK2, lentiviral vector expressing SIK2. **C**, Representative flow cytometry pictures of Annexin V/PI double-staining assays in SIK2-knockdown CRC cells 48 hours after IR. **D,** Assessment of SIK2 knockdown on apoptosis in HCT116 and HCT8 cells 48 hours after IR by WB. **E**, Representative images of IHC staining for KI67 and SIK2 in the harvested xenografts and H&E staining. Magnification, × 40. Scale bar, 50 μm. IR, ionizing radiation; non-IR, without ionizing radiation. $^{**}P < 0.01$; $^{***}P < 0.001$

Figure S3

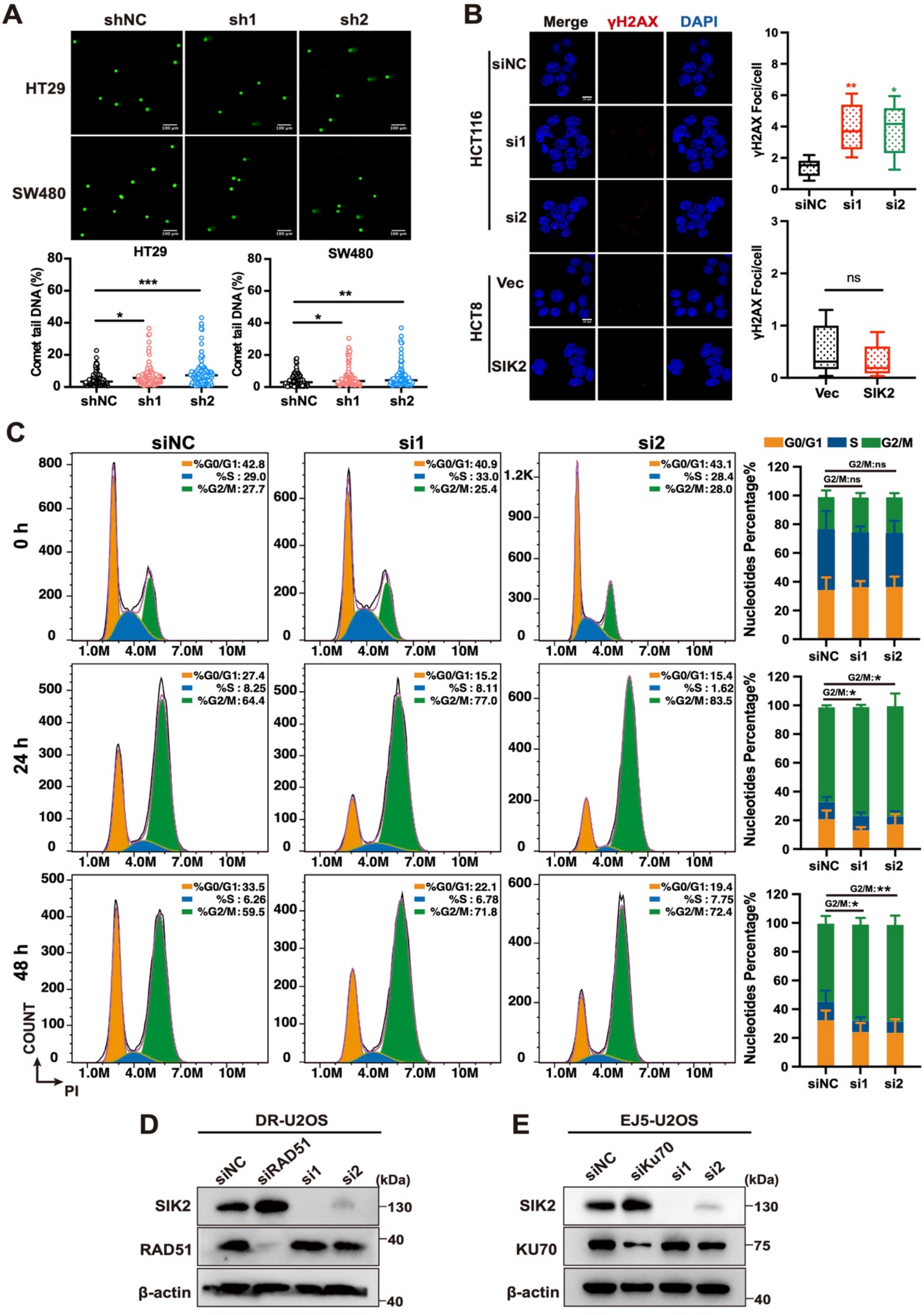

Figure S3. SIK2 promotes DNA damage repair in colorectal cancer. A, Comet assay was used to assess DNA damage in SIK2-knockdown CRC cells in the absence of IR (0 Gy). Upper,

representative pictures, scale bar, 100 μm; lower, spot charts indicating the percent of comet tail DNA, at least 100 cells were counted in each condition. **B**, Immunofluorescence was used to assess the effect of SIK2 expression on γH2AX foci in the absence of IR (0 Gy). Left, representative immunofluorescence images, scale bar, 10 μm; right, boxplots indicating the γH2AX foci numbers per cell, at least 100 cells were counted in each condition. **C**, Cell cycle assay was used to assess the effects of SIK2 knockdown in cell cycle distribution at indicated time points after IR. Left, representative images; right, quantification of nucleotides percentage of G0/G1, S, and G2/M; data are represented as mean ± SD of three biological replicates. **D-E**, DR-U2OS (**D**), and EJ5-U2OS (**E**) cells were transfected with indicated siRNAs, and then the whole cell lysates were detected by western blots. siNC, negative control; si1-2, SIK2 siRNAs; siRAD51 and siKU70, siRNAs targeting *RAD51* and *KU70* were used as positive controls. ns, not significant; *$P < 0.05$; **$P < 0.01$; ***$P < 0.001$.

Figure S4

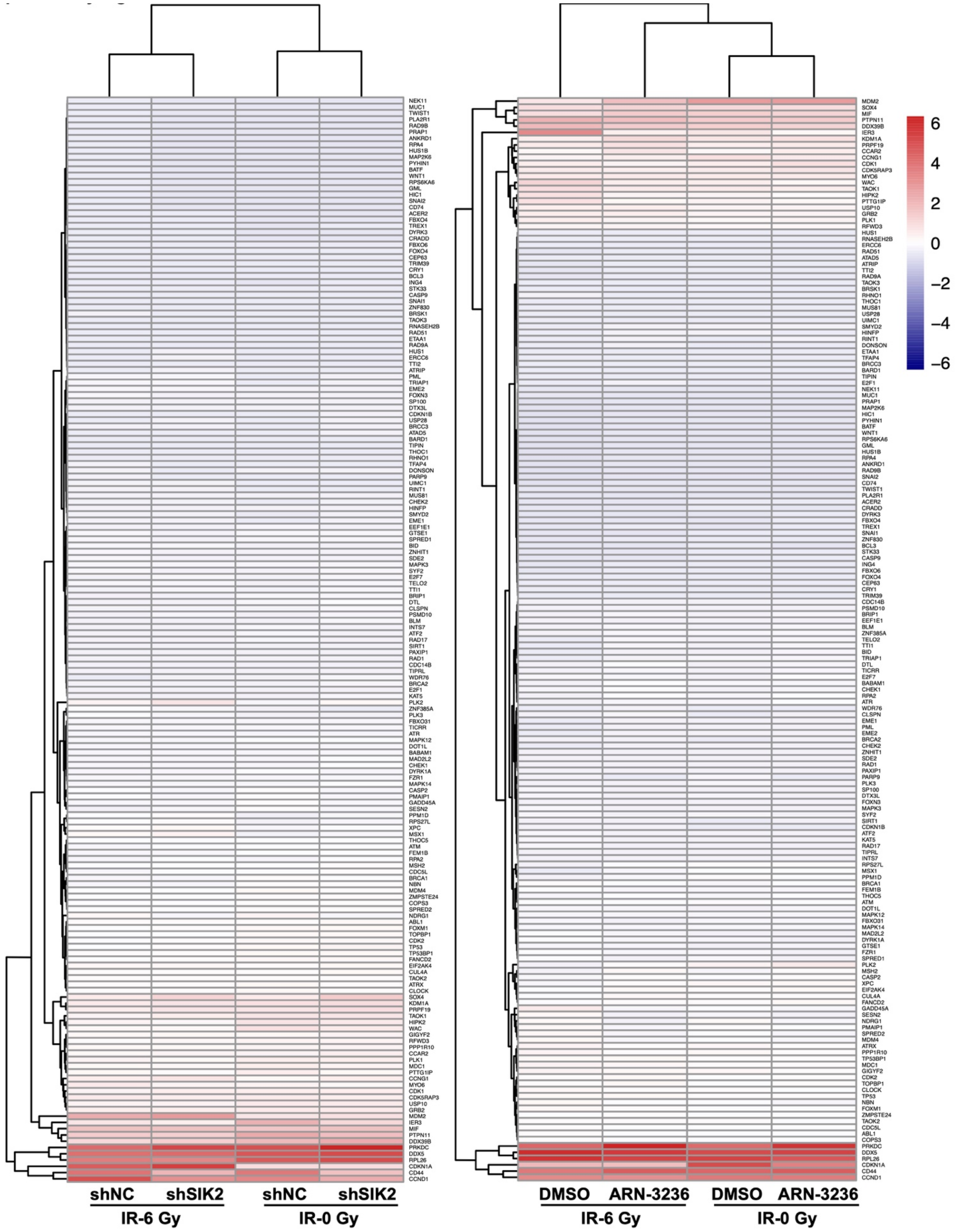

Figure S4. The heatmap presentation of DNA damage repair gene expression. HCT116 cells with SIK2 knockdown were exposed to indicated doses of X-rays and collected for mRNA

sequencing 48 hours after IR. The expressions of DNA damage repair genes are presented as color-coded heatmaps with red corresponding to upregulation and blue to downregulation. IR, ionizing radiation; non-IR, without ionizing radiation.

Figure S5

A

Gene names	MW [kDa]	Protein score	Sequence coverage (%)	Unique Peptides	Peptides
SIK2	103.8	1233	29	23	23
VCP	89.3	365	18	11	11
IGKV2-29	13.1	88	17	2	2
KHDRBS1	48.2	83	5	2	2
CSNK2A1	45.1	74	5	1	1
SLBP	31.3	70	10	2	2
RPL29	17.7	67	9	1	1
H2BC21	13.9	64	16	2	2
PIMREG	27.5	59	8	2	2
OTX1	37.3	58	3	1	1
HNRNPR	70.9	54	3	1	2
DROSHA	159.2	52	1	1	1
DNAJC9	29.9	51	5	1	1
UBA52	14.7	51	7	1	1
XRN2	108.5	50	1	1	1
TUBA1A	50.1	47	6	2	2
ZNF207	50.7	46	3	1	1
MCMBP	72.9	45	2	1	1
PNN	81.6	44	1	1	1
RNMT	54.8	44	2	1	1
ANKHD1	269.3	41	0	1	1
KLHL30-AS1	9.7	39	7	1	1

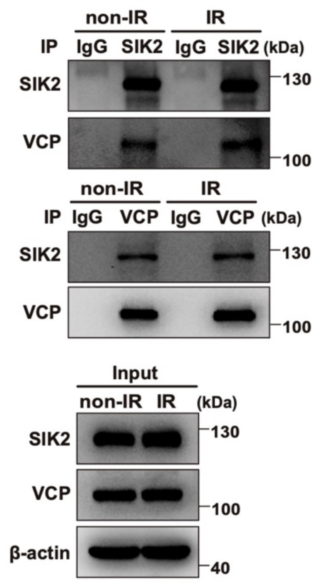

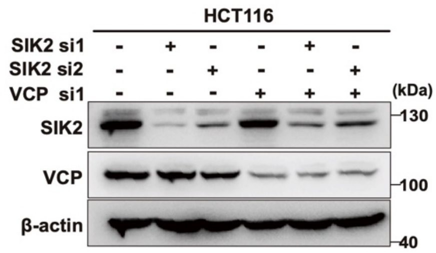

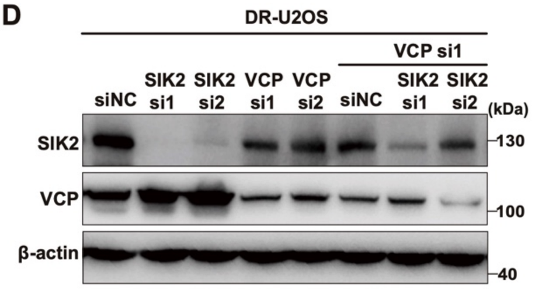

Figure S5. SIK2 promotes the radioresistance of CRC cells by interacting with VCP. A, List of proteins specifically interacting with SIK2 in HCT116 cells identified by the mass spectrometry. **B,** HCT116 cells were treated with IR (0 or 12 Gy) and harvested for Co-IP and WB assays. **C** and **D**, HCT116 (**C**), and DR-U2OS (**D**) cells were transfected with indicated siRNAs, and then the whole cell lysates were detected by western blots.

Figure S6

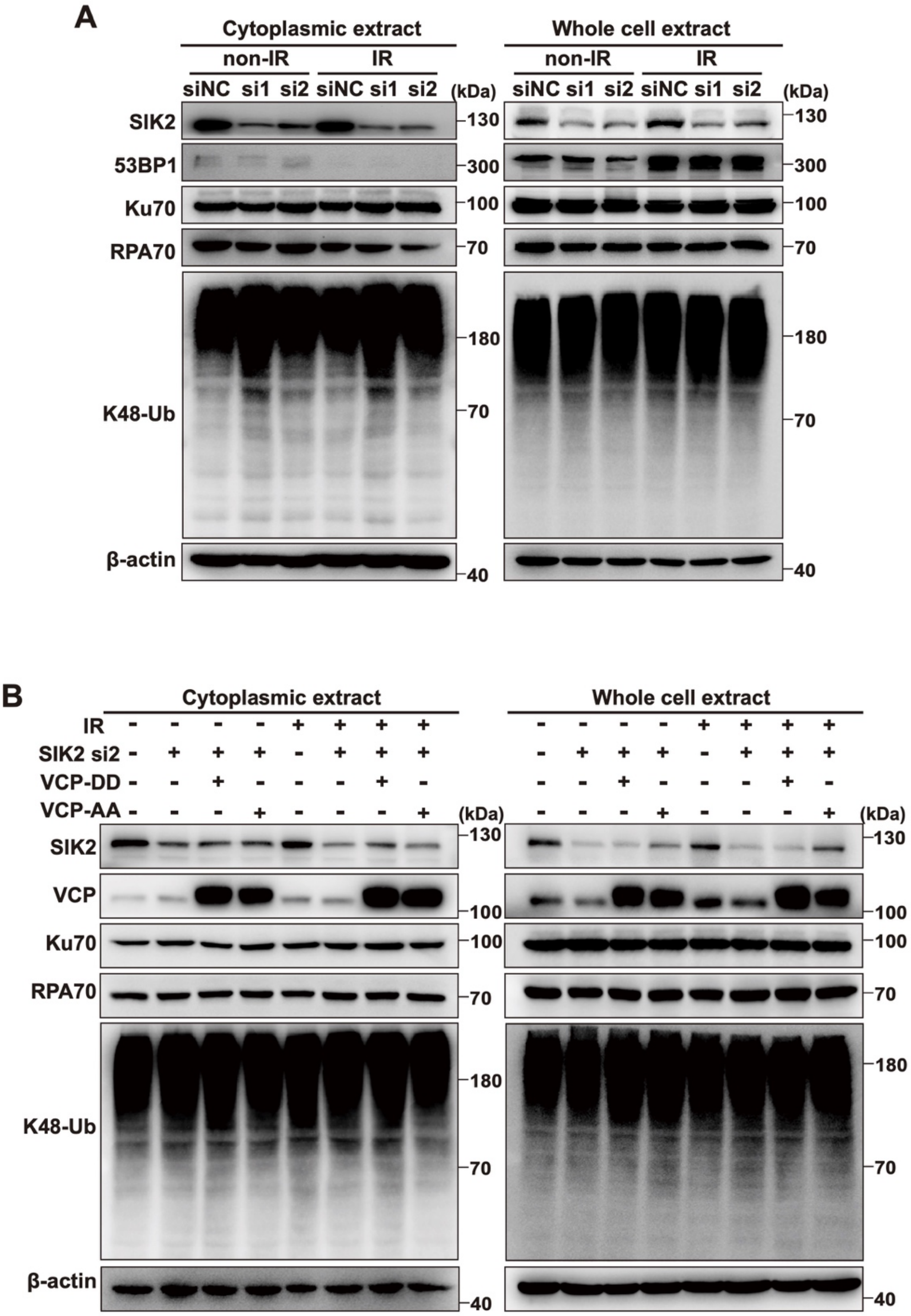

Figure S6. **A** and **B**, Western blots of the indicated proteins in subcellular fractions of HCT116 cells at 6 hours after IR (12 Gy). Cells were transiently transfected with siNC and *SIK2* siRNAs (**A**); siNC, negative control; si1-2, *SIK2* siRNAs. Cells with endogenous SIK2 silencing by siRNA after restoring VCP-DD or VCP- AA expression (**B**). IR, ionizing radiation; non-IR, without ionizing radiation.

Figure S7

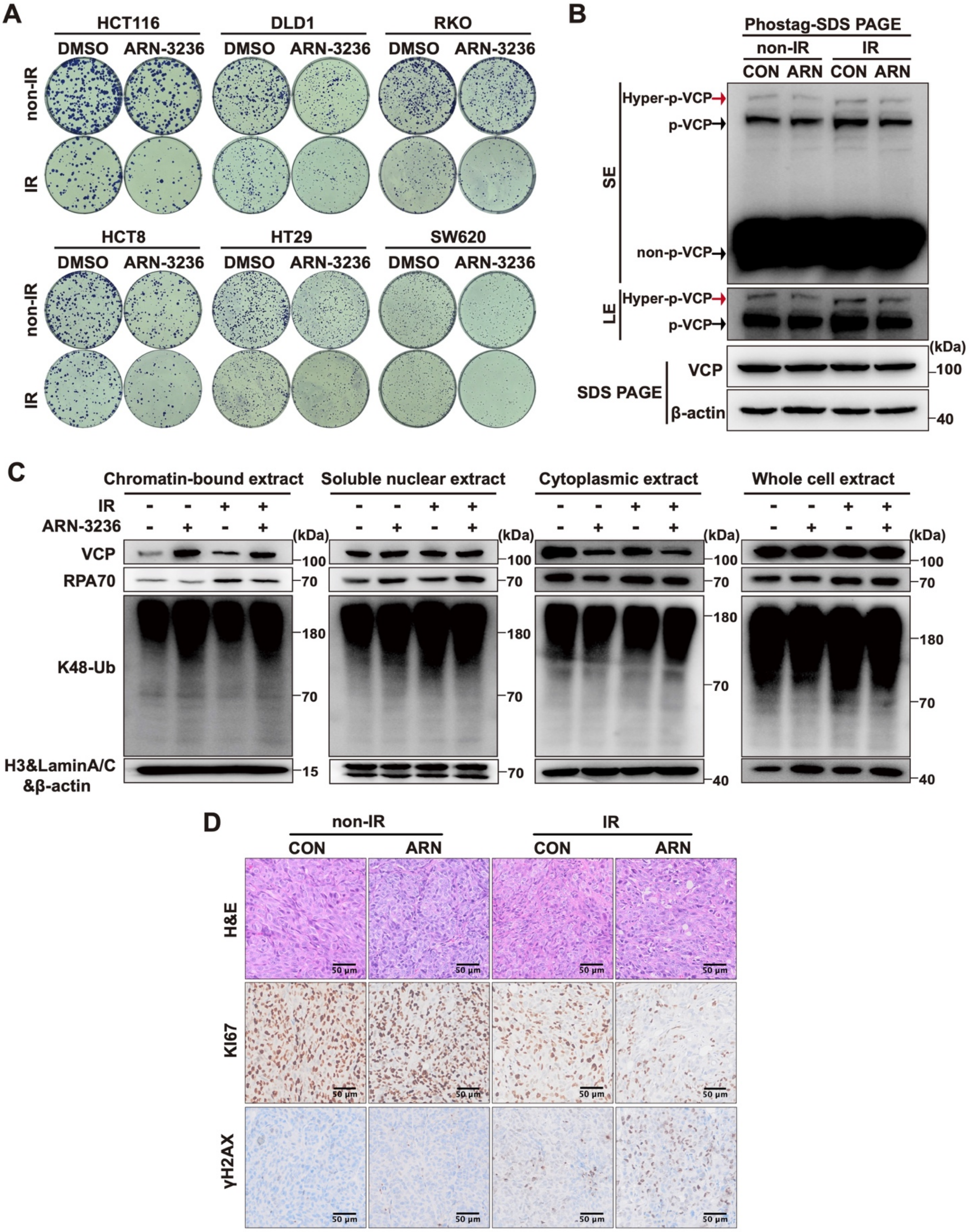

Figure S7. ARN-3236 significantly sensitizes CRC cells to radiation both *in vitro* and *in vivo*. A, Representative colony formation pictures of CRC cells treated with DMSO or ARN-3236. **B**, WBs (upper, Phos-tag^{TM}-SDS PAGE; lower, SDS PAGE) of the cell lysates at 6 hours

post-IR (12 Gy) from HCT116 cells treated with DMSO (CON) or 10 μM ARN-3236 (ARN). LE, long-term exposure; SE, short-term exposure; Hyper-p-VCP, hyperphosphorylated VCP; p-VCP, phosphorylated VCP; non-p-VCP, non-phosphorylated VCP. **C**, Western blots of the indicated proteins in subcellular fractions of HCT116 cells at 6 hours after IR; cells were treated selectively with ARN-3236 (10 μM) for 24 hours before IR (12 Gy). **D**, Representative images of IHC staining for KI67 and H&E staining in the harvested xenografts. Magnification, × 40. Scale bar, 50 μm. IR, ionizing radiation; non-IR, without ionizing radiation.

Figure S8

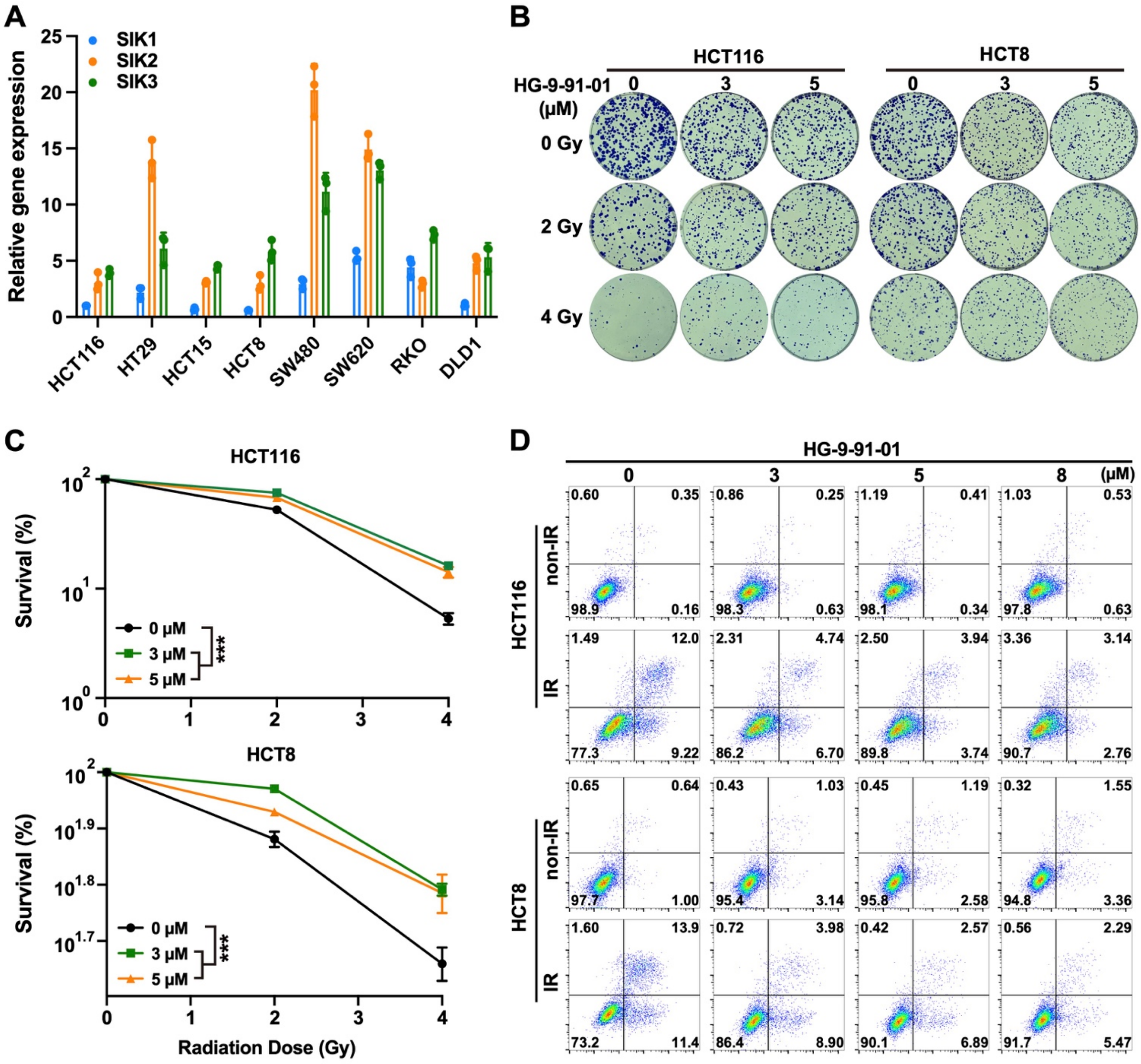

Figure S8. SIK2 plays a specific role in promoting radioresistance among the SIK family. **A**, Relative mRNA level of SIKs in CRC cells. **B-D**, Colony formation assays and flow cytometry-based Annexin V/PI double-staining assays were used to assess radiosensitivity and IR-induced apoptosis in CRC cells treated with indicated doses of HG-9-91-01. Representative colony formation pictures (**B**); the survival rates post-IR are presented as the mean ± SD (**C**). Representative pictures of flow cytometry (**D**). IR, ionizing radiation; non-IR, without ionizing radiation. ****P* < 0.001.

Table S1: candidate radioresistance genes selected in cells treated with 10Gy X-ray by MAGeCK analysis.

Gene	neg|score	neg|p-value	neg|rank	neg|good sgRNA	neg|LFC
CDKN1A	2.34E-06	1.48E-05	1	3	-0.64975
ADRBK1	9.88E-05	0.00038984	2	4	-1.265
CSNK2A2	0.0023933	0.0089169	3	4	-1.0135
PRKACG	0.0028239	0.0076142	4	2	-0.30076
COASY	0.0036642	0.013309	5	3	-0.85806
ROCK2	0.004155	0.014927	6	2	0.11105
CKS2	0.0051036	0.018105	7	3	-0.55377
IKBKE	0.0056133	0.015085	9	2	-0.77901
STK33	0.0066611	0.023247	11	4	-0.70287
MAP3K11	0.0067391	0.023445	12	4	-0.50302
EGFR	0.0072403	0.024945	13	2	0.12359
PFKM	0.0077725	0.026701	14	4	-0.5492
ADPGK	0.0086194	0.029435	15	2	-0.20271
AK1	0.0091238	0.031034	16	3	-0.14042
STK25	0.0093332	0.031705	17	3	-1.0979
NRBP2	0.0094149	0.031932	18	3	-0.90181
ROR2	0.010776	0.036127	20	2	-0.48329
PRKCZ	0.012426	0.041209	22	2	-0.084904
PRKCE	0.012438	0.041249	23	4	-0.35903
PLK1	0.01324	0.025379	25	2	-0.86082
PI4K2B	0.013792	0.045276	26	4	-0.5556
DDR2	0.014074	0.046075	27	3	-0.28422
DLG1	0.015248	0.049421	28	2	-0.33796
MAP3K7	0.01572	0.050822	30	2	-0.18487
DDR1	0.016597	0.053418	31	4	-0.1908
ERN1	0.016829	0.054069	32	4	-0.36105
PIKFYVE	0.017175	0.055145	34	2	-0.64586
EFNA5	0.017364	0.055727	35	2	-0.36027
PIM3	0.017424	0.055915	36	3	-1.4161
DCLK2	0.01746	0.055994	37	4	-0.15532
PRKCB	0.019843	0.036739	40	2	-0.46265
MST1R	0.019996	0.063336	41	3	-0.20984
HIPK4	0.020611	0.065113	42	4	-0.22445
AK3	0.020646	0.065241	43	2	-0.39548
CLK2	0.021115	0.066751	45	3	-1.0897
DGKA	0.022447	0.069712	48	3	-0.32623
DLG3	0.023865	0.07291	50	3	-0.29945
ULK3	0.023957	0.073117	51	3	-0.58697

PIP5K1B	0.025092	0.062794	53	3	-0.55077
CDKL2	0.026398	0.065794	56	3	-0.29683
DYRK4	0.026931	0.066919	57	2	-0.2352
EPHA7	0.027025	0.079394	58	3	-0.79451
EPHA3	0.027597	0.068695	61	2	-0.25873
STK24	0.027748	0.069021	62	3	-0.20532
CALM2	0.028496	0.070659	64	2	-0.44583
ERN2	0.029815	0.085286	67	3	-0.27271
STK40	0.031449	0.077252	70	3	-0.23066
DSTYK	0.03251	0.090674	71	2	0.17785
NME4	0.03368	0.092757	72	3	-0.42121
LMTK3	0.03369	0.092796	73	2	-0.23251
CIB2	0.033941	0.082591	74	2	-0.18867
SGK494	0.03784	0.1009	76	3	-0.60172
PRKAR2B	0.038655	0.093704	77	3	-0.23472
BRD2	0.039395	0.10394	78	2	0.0089335
CHEK2	0.039829	0.10487	79	3	-0.16491
ROCK1	0.040438	0.10615	80	3	-0.15191
MYLK	0.043387	0.11163	81	2	-0.021006
SIK2	0.04421	0.11336	82	3	-0.23195
CAMK4	0.045919	0.11088	84	3	-0.16796
CDK17	0.046617	0.11779	86	3	-0.39522
CDKL5	0.047098	0.084269	87	2	-0.24174
PKMYT1	0.049491	0.11818	88	2	-3.1511
MAPK3	0.049714	0.11868	89	2	-0.11459
IRAK4	0.051412	0.12657	91	2	-0.098643
ITPKC	0.053819	0.13148	93	3	-0.52453
PRKCA	0.05404	0.12816	94	3	-0.26792
AKT2	0.054421	0.13286	96	3	-0.26444
NME3	0.056936	0.13454	98	2	-0.4882

Table S2: candidate radioresistance genes selected in cells treated with 5Gy X-ray by MAGeCK analysis.

Gene	neg|score	neg|p-value	neg|rank	neg|good sgRNA	neg|LFC
SIK2	0.0010589	0.0041165	2	3	-0.51592
BMX	0.0019606	0.0074572	3	4	-0.7831
DGKK	0.0024066	0.0089595	4	4	-0.43975
UCKL1	0.0025507	0.0093548	5	3	-0.42836
PINK1	0.0036771	0.013338	6	3	-1.5314
HIPK4	0.0042485	0.015196	7	2	-0.1027
EPHA2	0.0051079	0.018151	8	3	-0.48657
PFKFB1	0.0059442	0.020919	10	2	-0.10807
CDK8	0.0063728	0.012656	11	2	-0.29738
MKNK1	0.0080882	0.027788	13	3	-0.23519
TSKS	0.009084	0.030931	16	4	-0.25428
DYRK2	0.0093289	0.031741	17	3	-0.81613
GRK7	0.0093801	0.03187	18	3	-0.8025
PKN3	0.0099613	0.033698	19	2	-0.17218
AK3	0.010347	0.034854	21	4	-0.36409
ATM	0.010736	0.020978	22	2	-0.41784
NIM1K	0.010853	0.036416	23	4	-0.30997
IKBKAP	0.012232	0.040607	24	3	-0.97411
NEK8	0.012925	0.033688	25	3	-0.66413
IPMK	0.013641	0.03525	26	3	-0.5127
AGK	0.014473	0.047061	27	3	-0.52722
SCYL1	0.015143	0.049215	28	3	-0.58575
CRKL	0.015545	0.040162	29	3	-0.28649
MAP3K13	0.015675	0.050727	30	2	-0.093334
MAGI3	0.016208	0.052259	32	4	-0.3337
PIK3R1	0.016781	0.054019	33	3	-0.24153
CLK2	0.017318	0.055679	35	4	-0.72563
CSK	0.017793	0.045539	36	3	-0.21273
CALM2	0.018397	0.047031	37	2	-0.27242
MARK2	0.018916	0.059672	38	3	-0.81012
PAK6	0.019405	0.049838	39	3	-0.33053
MAPKAPK2	0.019431	0.060908	40	2	-0.080487
PNCK	0.020795	0.063665	41	3	-0.45713
CDK16	0.021635	0.065266	43	3	-0.63233
TGFBR1	0.022102	0.056074	44	3	-0.17763
CDK12	0.022219	0.056351	45	3	-0.90194
GNE	0.022351	0.066808	46	3	-0.54507
LATS1	0.022633	0.057221	47	2	-0.37815
CDK11B	0.022781	0.067777	48	3	-0.34052
PRKAB2	0.027404	0.068123	50	3	-0.20887

TESK2	0.027791	0.07855	51	2	-0.00086939
PIM1	0.028224	0.069981	52	3	-0.44926
RYK	0.028443	0.080032	53	3	-0.50788
MARK1	0.031161	0.076395	57	3	-0.53449
ADCK5	0.03247	0.088097	58	3	-0.21685
PTK7	0.033096	0.080615	59	3	-0.48214
DAPK2	0.033762	0.090469	61	3	-0.36553
CAMK4	0.034429	0.083739	63	3	-0.10216
CSNK1D	0.03444	0.091912	64	3	-0.23764
TGFBR2	0.034697	0.08445	65	2	-0.42643
TAF1L	0.035922	0.087158	66	2	-0.76161
GRK4	0.036097	0.095253	68	3	-0.15147
ALDH18A1	0.036637	0.088858	69	3	-0.69794
DLG1	0.038024	0.098999	71	3	-0.30758
MAP2K3	0.038433	0.093276	72	2	-0.83931
KCNH2	0.038566	0.069556	73	2	-0.178
BCKDK	0.039446	0.10189	76	3	-0.36182
PRKDC	0.040421	0.097813	78	3	-0.28932
LRRK1	0.04108	0.10521	80	2	-0.21967
PFKFB4	0.042098	0.10203	81	3	-0.5684
FYN	0.044138	0.10674	83	3	-0.44333
CHUK	0.044499	0.11173	84	2	-0.20296
CSNK1A1	0.044872	0.10858	85	2	-0.30534
TAOK1	0.047112	0.11298	86	2	-0.213
ALK	0.048834	0.12001	88	2	-0.049752
DGKB	0.05127	0.12439	89	2	-0.34488
FN3K	0.051937	0.12191	90	2	-0.62636
FN3KRP	0.051987	0.12586	91	3	-0.23093
AURKC	0.052241	0.12235	92	2	-1.0856
DYRK4	0.053158	0.12373	93	2	-0.15277
FGFR1	0.054698	0.13159	96	3	-0.19587
JAK3	0.054703	0.12588	97	2	-0.26077
PGK2	0.056968	0.12908	98	2	-0.66377
DLG4	0.057751	0.13705	99	3	-0.30623
PAK2	0.05801	0.13752	100	3	-0.31338

Table S3: Detailed information about the sensitivity enhancement ratio of SIK2 knockdown in different CRC cells.

cell line	group	linear-quadratic model	D_0/Gy	SER
HCT116	shNC	Y=exp（-1*（0.2403*X+0.0888*X^2））	2.256	
	sh1	Y=exp（-1*（0.2698*X+0.2137*X^2））	1.616	1.396
	sh2	Y=exp（-1*（0.3786*X+0.1746*X^2））	1.537	1.468
	sh3	Y=exp（-1*（0.3684*X+0.1314*X^2））	1.686	1.339
HCT8	shNC	Y=exp（-1*（0.03778*X+0.05971*X^2））	3.776	
	sh1	Y=exp（-1*（0.1526*X+0.06636*X^2））	2.888	1.308
	sh2	Y=exp（-1*（0.1761*X+0.06264*X^2））	2.819	1.340
	sh3	Y=exp（-1*（-0.01091*X+0.1071*X^2））	3.098	1.219
SW480	shNC	Y=exp（-1*（0.1907*X+0.04665*X^2））	3.005	
	sh2	Y=exp（-1*（0.2736*X+0.07283*X^2））	2.267	1.326
	sh3	Y=exp（-1*（0.2754*X+0.06838*X^2））	2.298	1.307
HT29	shNC	Y=exp（-1*（-0.004039*X+0.0696*X^2））	3.809	
	sh2	Y=exp（-1*（0.2*X+0.0621*X^2））	2.703	1.409
	sh3	Y=exp（-1*（0.09568*X+0.1204*X^2））	2.504	1.521

D_0: Mean Lethal Dose. It indicates the radiation dose required to kill 63% of the cells in the fitted survival curve; **SER**: Sensitivity Enhancement Ratio; **X**: Radiation Dose; **Y**: Proportion of Surviving Cells.

Table S4: the sequences of siRNAs.

Target gene	Name	Direction	Sequence
Scramble	siNC	Sense	5'-GCGACGAUCUGCCUAAGAU-3'
		Antisense	5'-AUCUUAGGCAGAUCGUCGC-3'
RAD51	siRAD51	Sense	5'-AAGGGAAUUAGUGAAGCCAAA-3'
		Antisense	5'-UUUGGCUUCACUAAUUCCCUU-3'
Ku70	siKu70	Sense	5'-GAUGAGUCAUAAGAGGAUCAU-3'
		Antisense	5'-AUGAUCCUCUUAUGACUCAUC-3'
SIK2	SIK2 si1	Sense	5'-CCGUAUUUCAUGUCAGAAGAU-3'
		Antisense	5'-AUCUUCUGACAUGAAAUACGG-3'
	SIK2 si2	Sense	5'- GCUAACUGUAUCCCUAGAAAU-3'
		Antisense	5'- AUUUCUAGGGAUACAGUUAGC-3'
	SIK2 si3	Sense	5'-GCUCAUGCCUUUGAGGCAUUU-3'
		Antisense	5'-AAAUGCCUCAAAGGCAUGAGC-3'
VCP	VCP si1	Sense	5'-AACAGCCAUUCUCAAACAGAA-3'
		Antisense	5'-UUCUGUUUGAGAAUGGCUGUU-3'
	VCP si2	Sense	5'-AAGUAGGGUAUGAUGACAUUG-3'
		Antisense	5'-CAAUGUCAUCAUACCCUACUU-3'
	VCP si3	Sense	5'-CCUAGCCCUUAUUGCAUUGUU-3'
		Antisense	5'- AACAAUGCAAUAAGGGCUAGG-3'

Table S5: the sequences of primers in point mutation.

Name	Direction	Sequence
$SIK2^{K49M}$	Sense	5'-GGTGGCAATAATGATAATCGATAAG-3'
	Antisense	5'-CTTATCGATTATCATTATTGCCACC-3'
VCP^{S784A}	Sense	5'-TGGAGCTGGCCCCGCTCAGGGCAGTGGA-3'
	Antisense	5'-TCCACTGCCCTGAGCGGGGCCAGCTCCA-3'
VCP^{S784D}	Sense	5'-TGGAGCTGGCCCCGATCAGGGCAGTGGA-3'
	Antisense	5'-TCCACTGCCCTGATCGGGGCCAGCTCCA-3'
VCP^{S770A}	Sense	5'-GTCGGGGCTTTGGCGCCTTCAGATTCCCTTC-3'
	Antisense	5'-GAAGGGAATCTGAAGGCGCCAAAGCCCCGAC-3'
VCP^{S770D}	Sense	5'-GTCGGGGCTTTGGCGACTTCAGATTCCCTTC-3'
	Antisense	5'-GAAGGGAATCTGAAGTCGCCAAAGCCCCGAC-3'

Table S6: the information of shRNAs.

Target gene	Name	Position	Target sequence
Scramble	shNC	/	5'-GCGACGATCTGCCTAAGAT-3'
SIK2	SIK2 sh1	CDS	5'-CCGTATTTCATGTCAGAAGAT-3'
	SIK2 sh2	3'-UTR	5'-GCTAACTGTATCCCTAGAAAT-3'
	SIK2 sh3	CDS	5'-GCTCATGCCTTTGAGGCATTT-3'
DYRK4	shDYRK4	CDS	5'-GTACATCCAAAGCCGGTTCTA-3'
CAMK4	shCAMK4	CDS	5'-AGAAAGTTAAAGGTGCAGATA-3'
HIPK4	shHIPK4	CDS	5'-CGCTTCCTTGAGTTCTTCCAT-3'
DLG1	shDLG1	CDS	5'-CGGGTCAATGACTGTATATTA-3'
AK3	shAK3	CDS	5'-ATTCAGCGTGAGGATGATAAA-3'

CDS: coding DNA sequence; 3'-UTR: 3'-untranslated region.

Table S7: the sequences of real-time PCR Primers.

Target	Direction	Sequence
SIK1	Forward	5'-TTCTCCGCACACAGCTACAC-3'
	Reverse	5'-TTTGCAGTGACTCCACCGTC-3'
SIK2	Forward	5’-AGACCACCCTCACATAATCAAAC-3’
	Reverse	5’-ATTTTCGCCTGGCTTCAGACT-3’
SIK3	Forward	5'-TACAGCCTGCTGTGTGATCG-3'
	Reverse	5'-TGCTCCGCCTGGATATTGAC-3'
DYRK4	Forward	5'-TCCACCCTAGCATTAAAACCCA-3'
	Reverse	5'-CGTAGCCCAGGATTTCACTTT-3'
CAMK4	Forward	5'-GCCTCGTCCCGGATTACTG-3'
	Reverse	5'-TCCCCTTCTGTTTGCATCTGT-3'
HIPK4	Forward	5'-ACTGACTGCTACGACATCATCG-3'
	Reverse	5'-GGTAGGCGTCATTCTTGAGGAT-3'
DLG1	Forward	5'-GCAGGAGGTACGGACAACC-3'
	Reverse	5'-ATTGACCCGCAATCTTCCATC-3'
AK3	Forward	5'-GGCAGAAGCCCTAGATAGAGC-3'
	Reverse	5'-GCAGTAAGGCGTTGTTTAATGAC-3'
GAPDH	Forward	5’-GAAGGTGAAGGTCGGAGTC-3’
	Reverse	5’-GAAGATGGTGATGGGATTTC-3’

Table S8: the information of primary antibodies.

Antibody	Source	Cat. #	Application and dilution
SIK2	Santa Cruz	sc-393139	WB 1:500
ATM	Santa Cruz	sc-377293	WB 1:500
SIK2	Abcam	ab53423	IHC 1:500
VCP	Abcam	ab109240	WB 1:10000
RAD51	Abcam	ab133534	WB 1:10000 IF 1:200
Histone H3	Abcam	ab1791	WB 1:5000
Lamin A/C	Abcam	ab108595	WB 1:10000
Lamin B1	Abcam	ab16048	WB 1:1000
RPA70	Abcam	ab79398	WB 1:1000
GAPDH	Proteintech	HRP-60004	WB 1:20000
β-actin	CST	3700	WB 1:2000
γH2AX(Ser139)	CST	9718	WB 1:1000 IHC&IF 1:200
KU70	CST	4588	WB 1:1000
K48-Ub	CST	8081	WB 1:1000
p-ATM(Ser1981)	CST	5883	WB 1:1000
cleaved PARP	CST	5625	WB 1:1000
53BP1	CST	4937	WB 1:1000
Myc(tag)	CST	2276	WB 1:1000
Flag	CST	8146	WB 1:1000
KI67	ZSGB-BIO	ZM-0167	IHC 1:200

CST: Cell Signaling Technology.
